# Targeting ribosomes reprograms the tumour microenvironment and augments cancer immunotherapy

**DOI:** 10.1038/s41416-025-03109-y

**Published:** 2025-07-11

**Authors:** Kaisa Cui, Bingxin Liu, Liang Gong, Quan Wan, Hong Tang, Zhicheng Gong, Renhui Shen, Chao Wang, Qiang Zhang, Qilin Li, Yizhun Zhu, Youming Zhang, Xiaojie Lu

**Affiliations:** 1 Wuxi Cancer Institute, Affiliated Hospital of Jiangnan University, Wuxi, Jiangsu 214062 China; 2 Neuroscience Center, Wuxi School of Medicine, Jiangnan University, Wuxi, Jiangsu 214122 China; 3 The Key Laboratory of Modern Toxicology of Ministry of Education, Center for Global Health, School of Public Health, Nanjing Medical University, Nanjing, Jiangsu 211166 China; 4 Key Laboratory of Quantitative Synthetic Biology, Shenzhen Institute of Synthetic Biology, Shenzhen Key Laboratory of Genome Manipulation and Biosynthesis, Shenzhen Institutes of Advanced Technology, Chinese Academy of Sciences, Shenzhen, Guangdong 518055 China; 5 Department of Neurosurgery and Emergency Medicine, Jiangnan University Medical Center, Wuxi, Jiangsu 214002 China; 6 Department of Pathology, Jiangnan University Medical Center, Wuxi, Jiangsu 214002 China; 7 Department of General Surgery, The Affiliated Wuxi People’s Hospital of Nanjing Medical University, Wuxi, Jiangsu 214002 China; 8 Department of Gastrointestinal Surgery, Affiliated Hospital of Jiangnan University, Wuxi, Jiangsu 214122 China; 9 Department of Biochemistry, Molecular Cancer Research Center, School of Medicine, Shenzhen Campus of Sun Yat-sen University, Shenzhen, Guangdong 518107 China; 10 Computer Vision Lab, Department of Electrical Engineering, California Institute of Technology, Pasadena, California 91125 USA; 11 School of Pharmacy and State Key Laboratory of Quality Research in Chinese Medicine, Macau University of Science and Technology, Macau, China

**Keywords:** Cancer microenvironment, Immunotherapy, Tumour immunology

## Abstract

**Background:**

Hyperactive ribosome biogenesis is a hallmark of tumours. Current ribosome-related studies are concentrated on cancer cells. Ribosomes can regulate both tumour and non-cancer cells within the tumour microenvironment, yet the immunomodulatory effects of cellular ribosome biogenesis blockade remain inadequately understood.

**Methods:**

We performed ribosome-targeting therapy utilizing CX-5461, an effective and acknowledged selective inhibitor of ribosome biogenesis, in immunocompetent in vivo models and submitted for single-cell RNA sequencing (scRNA-seq). Additional large-scale human scRNA-seq data, in-house clinical samples and assays were used.

**Results:**

Ribosome inhibition elevated lymphoid cell cytotoxic granule secretion and macrophage pro-inflammation reprogramming. We uncovered unique immune cell subpopulations that are sensitive to ribosome biogenesis blockade and are associated with adverse clinical outcomes. Impressively, these cells regress during responsive immune checkpoint blockade (ICB) treatment, revealing that they are essential for immunotherapy efficacy. Moreover, targeting ribosomes induces immune checkpoint expression (such as Lag3) and significantly sensitizes tumours to anti-Lag3 immunotherapy, eliciting potent tumour regression and deeper anti-tumour immune responses.

**Conclusions:**

These findings unravel previously unrecognized roles of cellular ribosome biogenesis in sustaining immunosuppressive non-cancer cells. Our work unveils that ribosome biogenesis blockade could reinstate immunosurveillance and provide novel strategies to enhance the ICB efficacy in patients with poor immunogenicity.

## Background

Ribosomes are among the oldest molecular machines in extant life and translate information from mRNAs into functional proteins within cells [1, 2]. Human ribosomes consist of a 40S small subunit (18S ribosome RNA [rRNA] and ribosomal S-proteins) and a 60S large subunit (5S/5.8S/28S rRNA and ribosomal L-proteins) [2]. Ribosome biogenesis starts with RNA polymerase I (Pol I) which generates 5.8S/18S/28S rRNAs. 5S rRNA and transfer RNA (tRNA) substrates used in new protein synthesis are transcribed by polymerase III [3]. Hyperactive ribosome biogenesis in cancer cells is a hallmark of tumour initiation and progression [1, 4]. Accordingly, current tumour ribosome-related studies, such as our previous studies [5, 6], are concentrated on cancer cells. However, the relationship between ribosome biogenesis and non-cancer cells is a less understood area of research.

Numerous less genotoxic drugs have been identified and developed to selectively target ribosome biogenesis, with these drugs increasing in value in clinical investigations [1, 7]. CX-5461 (Pidnarulex) is an effective and acknowledged small molecule selective inhibitor of Pol I, and is the “first‐in‐class” selective inhibitor of ribosome biogenesis [7–10]. It has been tested in phase I and II trials, such as NCT02719977 and NCT04890613. A previous study on in vitro cancer cell lines and in vivo patient-derived xenograft models revealed that CX-5461 is a promising therapy in combination with a PARP inhibitor in ovarian cancer [9]. Interestingly, a recent study revealed that CX-5461 combined with anti-PD-1/PD-L1 enhances therapeutic efficacy in colorectal cancer (CRC) [11]. The tumour microenvironment (TME) is a complex ecosystem of diverse cell types that supports to tumourigenesis and modulates resistance to different therapies. Accordingly, aside from cancer cells, certain non-cancer cells that have relatively active ribosome biogenesis are present in the TME and are sensitive to ribosome-targeting therapy. Understanding how ribosome biogenesis blockade reshapes the TME may assist in the rational development of new therapeutic approaches.

Single-cell RNA sequencing (scRNA-seq) technologies, such as those utilized in our previous studies on gastrointestinal cancers [12–14], have improved our understanding of cancers by characterizing transcriptomes at a cellular resolution, and identifying cell types and their expression profiles. Herein, we combined experimental mouse models and in-house clinical samples of solid tumours to decipher the impact of global ribosome biogenesis dysregulation on the TME. Ribosome-targeting therapy was performed utilizing CX-5461 in tumour murine models, generating and characterizing the single-cell atlas of pre- and post-treatment samples. To locate resultant pivotal cell subpopulations sensitive to ribosome-targeting inhibition, the level of cellular ribosome biogenesis needs to be determined, which is difficult to define directly in large numbers of single cells. Thus, we used the non-negative matrix factorization (NMF) approach to learn coordinated expression programs consistent with intertumoural ribosome biogenesis signaling at the single-cell resolution [13, 15], developing a general signature to characterize the ribosome biogenesis of individual cells within the TME. Computational and experimental assays were performed to further identify unique ribosome biogenesis active cell subpopulations that play causal roles in TME reprogramming and immunotherapeutic-resistance. Finally, we revealed that ribosome-targeting inhibition treatment enhances immune checkpoint blockade (ICB) efficacy in neoplasia.

## Results

### Targeting ribosomes alters the TME

**Fig. 1:**
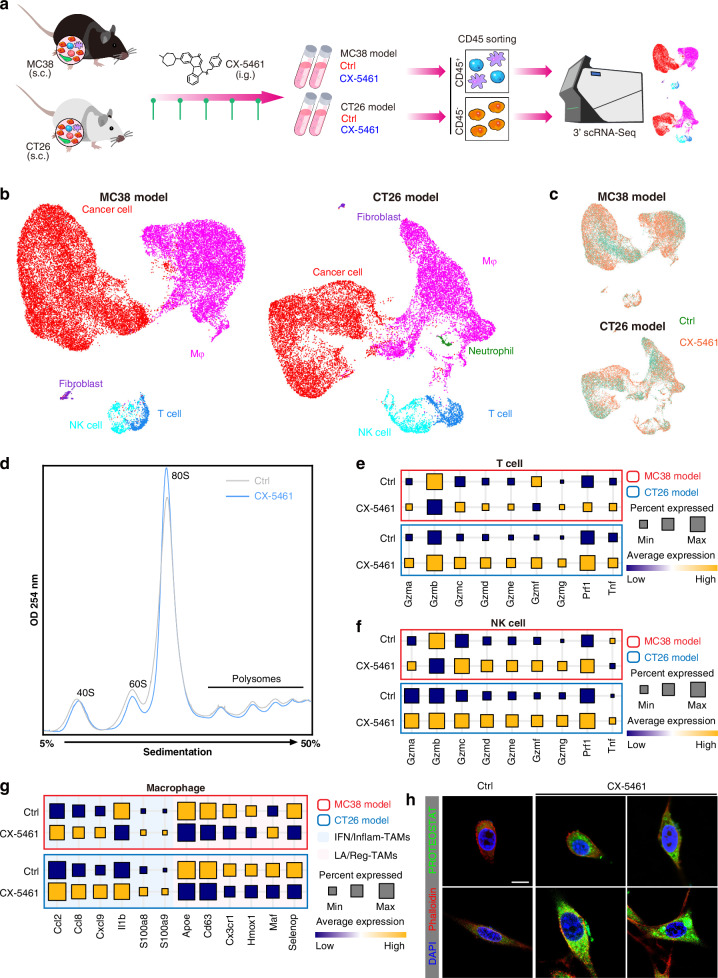
Targeting ribosome alters the TME. **a** Schematic showing therapy experiments of CX-5461 administration in tumor murine models. **b**, **c** UMAP plots showing cell types (**b**) and treatment groups (**c**) of CX-5461 administrated tumor murine models. **d** Polysome fractionation assays of CT26 cells extract without or with CX-5461 administration. **e, f** Dotplot showing expressions of cytotoxic granules in T (**e**) and NK (**f**) cells of CX-5461 administrated murine models. Dot size indicates proportion of cells expressing genes, dot color indicates mean expression of genes. **g** Dotplot showing expressions of TAM transcriptional states marker in macrophage of CX-5461 administrated tumor murine models. **h** Immunostaining of misfolded proteins marker PROTEOSTAT (green) and cytoskeleton marker Phalloidin (red) in CT26 cells upon CX-5461 treatment. Scale bar =10 µm.

To search for the TME alternation during the ribosome biogenesis blockade, we constructed MC38 and CT26 derived syngeneic  tumour models in immunocompetent mice. Then, we profiled the TME upon ribosome biogenesis blockade via scRNA-seq (Fig. 1a). After quality control, we recovered a total of 28,380 and 20,505 cells from the MC38 and CT26 murine models, respectively (Fig. 1b, c). Major cell types were annotated based on marker genes (Fig. S1a). Gene set enrichment analysis (GSEA) of cancer cells from syngeneic tumours revealed that ribosome biogenesis was drastically decreased upon CX-5461 treatment (Fig. S1b left). Additional polysome fractionation assays further showed that CX-5461 inhibited the 60S subunit and polysomes in both mouse and human cancer cells (Figs. 1d and S1c). A previous study suggested that CX-5461 activates the DNA damage response in the ovarian cancer cell lines in vitro [9]. Surprisingly, GSEA analysis of cancer cells from syngeneic tumours revealed that the DNA damage response was unchanged upon CX-5461 treatment (Fig. S1b right). Immunostaining confirmed that CX-5461 treatment had almost no effect on DNA damage in cancer cells, whereas treatment with 5-fluorouracil (5-FU), a known inducer of DNA damage in cancer cells [16], strongly increased the level of DNA damage markers (Fig. S1d). Hence, CX-5461-induced DNA damage likely occurs in a certain subpopulation of cancer cells, and CX-5461 is believed to be a bona fide inhibitor of ribosome biogenesis.

T and NK cells play central roles in the anti-tumour response and immunotherapy [17]. Of note, CX-5461 treatment directly activated T and NK cells, as evidenced by the levels of the most T and NK cells-secreted cytotoxicity factors, perforin and cytokine exhibiting the increase (Fig. 1e, f). Tumour-associated macrophages (TAMs) exhibit complex phenotypes, thereby the transcriptomic diversity of TAMs, such as interferon-primed TAMs (IFN-TAMs, high expression of IFN-regulated genes and M1-like markers), inflammatory cytokine-enriched TAMs (Inflam-TAMs, enriched with inflammatory cytokines), lipid-associated TAMs (LA-TAMs, characterized by canonical M2-like pathways, fatty acid metabolism and immunosuppression) and immune regulatory TAMs (Reg-TAMs, high expression of T cell suppression and immune checkpoints [ICs]), has been reviewed via scRNA-seq cancer studies [18]. We found CX-5461 treatment induced representative marker expression of IFN/Inflam-TAMs, and inhibited marker expression of LA/Reg-TAMs (Fig. 1g). Thus, the CX-5461-treated TME is likely immunogenic.

High microsatellite instability cases could benefit from immunotherapy at least in part because these cancer cells probably harbor many somatic mutations that encode potential neoantigens, and are therefore likely to be immunogenic, as well as inducing ICs upregulation [19]. The final event of ribosome biogenesis is new protein synthesis. Does CX-5461 administration directly lead to cancer cell mistranslation, resulting in the production of misfolded proteins? Interestingly, considerable accumulation of misfolded proteins was observed intracellularly. Notably, accumulated misfolded proteins are also present outside the membrane, suggesting the formation of unique tumour-specific neoantigens (Fig. 1h).

This initial evidence suggests that blockade of ribosome biogenesis likely disrupted the tumour permissive microenvironment by regulating lymphoid (T and NK) cells and macrophages. Targeting ribosomes leads to the production of misfolded proteins on the surface of cancer cells, probably as unique neoantigens that increase immunogenicity in the TME.

### Defining cellular ribosome biogenesis levels

**Fig. 2:**
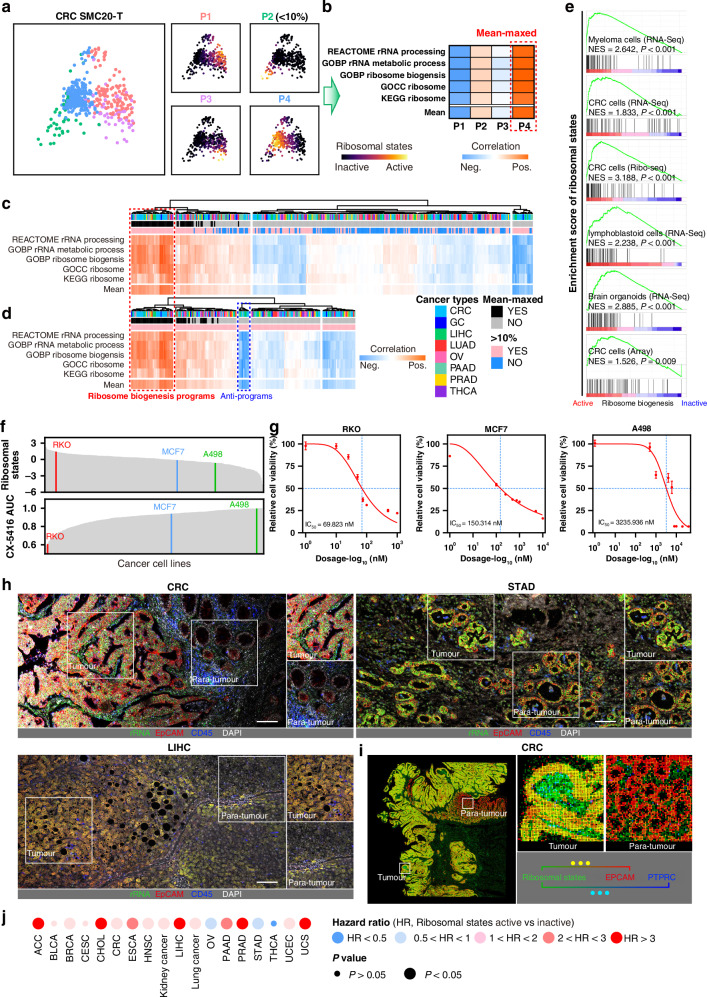
Defining cellular ribosome biogenesis levels. **a**, **b** Schematic representation of the analysis strategy to identify ribosome-related programs. In this tumor, UMAP plots showing each NMF program and its program score (**a**) heatmaps showing correlation coefficients between NMF program score and ribosome signature scores (**b**). **c**, **d** Hierarchically clustered heatmaps showing all correlation coefficients between NMF program scores and ribosome signature, including (**c**) and excluding (**d**)  ≤ 10%-programs. **e** GSEA plots showing the enrichment of ribosome biogenesis levels in samples from independent GEO datasets. **f** Spikes plots showing ribosomal states (Up) and AUC (bottom) within cancer cell lines of the Depmap. RKO, MCF7 and A498 cells were selected represent relative higher, middle and lower ribosome biogenesis activity cell lines used in our drug sensitivity validation. **g** IC_50_ curves of CX-5461 for RKO, MCF7 and A498 cell lines. **h** Multi-color immunostaining of rRNA (Y10B), epithelial cells (EPCAM) and immune cells (PTPRC/CD45) in tissues of CRC, STAD (stomach adenocarcinoma) and LIHC (hepatocellular carcinoma). Scale bar for all immunostaining images = 100 µm. **i** Pseudo immunofluorescence based on Visium HD images of CRC showing the spatial organization of spots assigned to cellular ribosome biogenesis levels, epithelial (EPCAM), stromal (DCN) and immune (PTPRC/CD45) cell markers. **j** Dotplot showing associations of ribosome biogenesis levels with patient overall survival in cancer types from the TCGA. Dot size indicates *P* values, dot color indicates HR values.

To investigate cell subpopulations that are sensitive to ribosome-targeting inhibition, large-scale single-cell transcriptomes are needed to develop a robust approach for evaluating the level of cellular ribosome biogenesis. Therefore, droplet-based integrated scRNA-seq data of eight solid tumour types were collected following rigorous quality control (Fig. S2a–c) [20–27]. After improving data integration and removing batch effects, nearly 500,000 cells were annotated based on canonical markers (Fig. S2d, e). Five current ribosomal signatures were subsequently used, and these gene sets were variable, with more than 40% and 20% of genes present in only single and double signatures, respectively [28]. The NMF approach was utilized to characterize ribosome-related cell programs in tumour epithelial cells [13, 15]. We correlated the NMF program score with five ribosomal signature levels in each tumour (Fig. 2a). We defined the program that showed the most positive correlation level in each tumour as mean-maxed program (Fig. 2b). Overall, 348 NMF programs were identified, containing 158 programs whose cell numbers were no more than 10%. We identified those that were well correlated with ribosome signature levels via hierarchical clustering programs (Fig. 2c, d). Specifically, 37 mean-maxed programs were clustered together regardless of whether these ≤10% cell-programs were included, suggesting that this intratumoural ribosome biogenesis active cluster is robust, specific and readily captured in scRNA-seq. Accordingly, programs of this cluster were defined as ribosome-related cell programs.

Next, each ribosome-related cell program was summarized by the top 200 genes based on NMF coefficients, and 2983 genes that were present in at least one program were obtained. Assessing the frequency at which each gene was expressed (Fig. S3a, b), a general signature was assembled from genes that were present in ten or more programs, establishing a ribosomal state signature that contained 73 metagenes to characterize ribosome biogenesis activity (Fig. S3c). As a test-negative control, we defined a cluster of programs that showed the most negative correlation level as anti-programs (Fig. S3d). A total of 124 genes were expressed in more than two anti-programs and did not overlap with 73 metagenes (Fig. S3e). 73-metagenes ribosomal states were highly correlated with ribosomal assembly, rRNA/tRNA processes and translational activity (Fig. S3f). We observed that cancer cells with active ribosome biogenesis levels presented significant enrichment of ribosomal states compared with cells with inactive ribosome biogenesis levels (Fig. 2e). Moreover, the ribosomal state activity of three cancer cell lines, RKO, MCF7 and A498, decreased gradually. The dose-response curve (AUC) of CX-5461 was increased gradually in RKO, MCF7 and A498 cells (Fig. 2f). In line with these findings, our drug sensitivity assays showed that the IC_50_ increased gradually in RKO, MCF7 and A498 cells (Fig. 2g). These results indicate that the ribosomal state signature is robust and specific for characterizing cellular ribosome biogenesis activity.

We analysed the ribosomal state in tumourigenesis process. In the inducible CRC mouse model, control samples could be distinguished from CRCs by ribosomal state metagene expression patterns (Fig. S3g). Clinically, we detected the level of cellular ribosome biogenesis in multiple tumour types via ribosomal antibody Y10B staining. This is an anti-rRNA antibody that recognizes ribosomal epitopes containing 5.8S, 18S and 28S rRNA [6, 29–31]. Compared with the para-tumour regions, tumour regions presented greater rRNA levels, although the histomorphological features varied among these cancers (Fig. 2h). We also observed similar results in the spatial transcriptomics (ST, including Visium HD and Visium) data (Figs. 2i and S3h). Notably, some spots in tumour regions that contained potential immune cells demonstrated a more active ribosomal state according to the ST data. Moreover, the ribosomal state was associated with reduced survival and remained an independent prognostic variable in multiple solid tumours (Figs. 2j and S4a). Associations of a more active ribosomal state correlated with grade and stage progression in some cancers (Fig. S4b, c). Interestingly, increased ribosomal state was association with smoking cases in ESCA, HNSC and LUAD (Fig. S4d), which are primary sites that could be influenced by smoking via nicotine exposure [32]. Two single-cell epithelial cell subgroup intrinsic-consensus molecular subtypes (iCMS2-3) have been identified in CRC [33]. The ribosomal state was upregulated in iCMS2 epithelial cells, which were characterized by crypt gene expression and WNT/MYC activation (Fig. S3i, j). Interestingly, a previous study showed that the patterns of ribosomal protein (RP) genes could be used to show differences between primary tumour and normal tissues [34]. Similarly, control samples could be distinguished from CRCs by their RP gene expression patterns (Fig. S3k). The CRC Visium data hints that tumour regions presented higher RP scores than paratumour regions did (Fig. S3l). Overall, these data suggest that the cellular ribosome biogenesis (the ribosomal state) is associated with pro-tumourigenic microenvironments and a poor prognosis.

### HSP70^+^ CD8^+^ T cells are sensitive to ribosome-targeting therapy in tumours

**Fig. 3:**
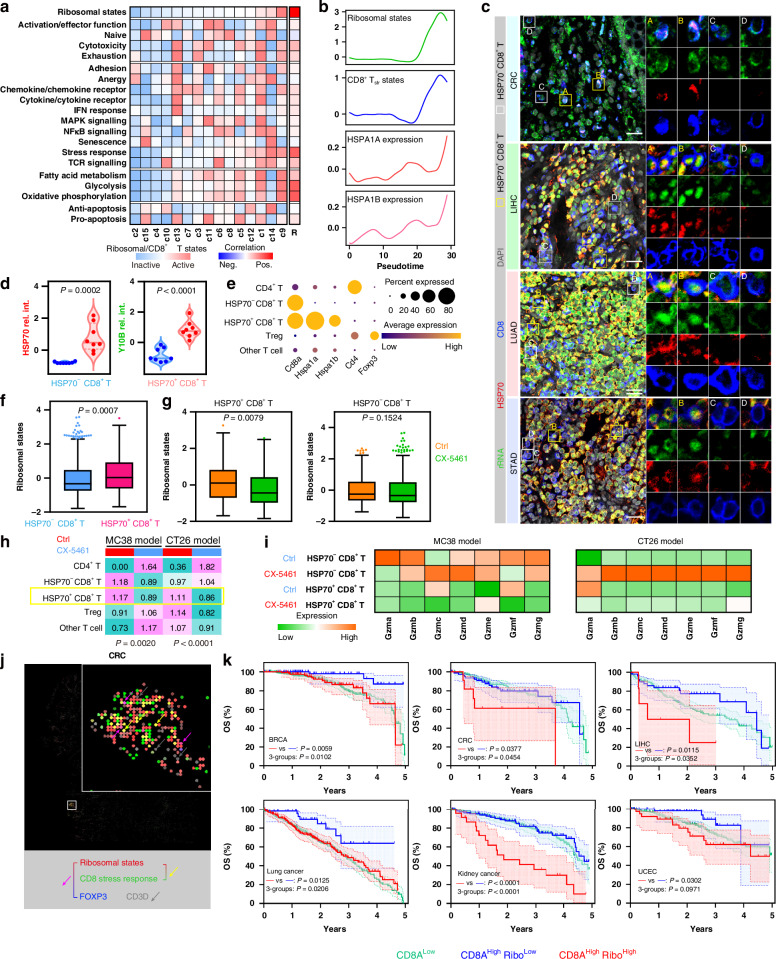
Tumor HSP70^+^ CD8^+^ T cells are sensitive to ribosome-targeting therapy. **a** Heatmap showing the cellular ribosome biogenesis and 19 curated CD8^+^ T cell states across CD8^+^ T cell clusters. Right single- column heatmap showing the correlation levels between cellular ribosome biogenesis and 19 CD8^+^ T cell states. **b** Expression pattern of cellular ribosome biogenesis, CD8^+^ T_str_ state, HSPA1A and HSPA1B along pseudotime in the trajectory of CD8^+^ T cells. **c**, **d** Multicolor-immunostaining of rRNA (Y10B), CD8^+^ T cells (CD8) and stress response marker (HSP70) in tissues of CRC, LIHC, LUAD (lung adenocarcinoma) and STAD (**c**). Scale bar for all immunostaining images = 20 µm. ROIs A and B represent HSP70^+^ CD8^+^ T (T_str_), C and D represent HSP70^-^ CD8^+^ T cells, (**d**) showing the intensity of HSP70 and rRNA levels for each ROI grouped by HSP70^+^ CD8^+^ and HSP70^-^ CD8^+^ T cells. **e** Dotplot showing expressions of marker genes of each T cell subtype of tumor murine models. Dot size indicates proportion of cells expressing genes, dot color indicates mean expression of genes. **f** Boxplots showing cellular ribosome biogenesis grouped by HSP70^+^ CD8^+^ T cells and HSP70^-^ CD8^+^ T cells of tumor murine models. **g** Boxplots showing cellular ribosome biogenesis in HSP70^+^ CD8^+^ T cells and HSP70^-^ CD8^+^ T cells of tumor murine models grouped by ctrl and CX-5461 treatment. **h** Heatmaps showing tissue prevalence of each T cell subtype estimated by Ro/e score grouped by ctrl and CX-5461 treatment of tumor murine models. **i** Heatmaps showing marker genes expression of granzymes in HSP70^+^/HSP70^-^ CD8^+^ T cells of CX-5461 administrated tumor murine models. **j** Pseudo immunofluorescence based on Visium HD images of CRC showing the spatial organization of spots assigned to cellular ribosome biogenesis levels, CD8^+^ T cell stress response state, Tregs (FOXP3) and T cell (CD3D) markers. **k** Kaplan–Meier plots showing the overall survival for each subgroup of the CD8^+^ T cell infiltration stratified by ribosome biogenesis levels within BRCA (breast carcinoma), CRC, LIHC, lung cancer, kidney cancer and UCEC (endometrial carcinoma) of the TCGA.

Next, we utilized the cellular ribosomal state to decipher non-cancer cell subpopulations that sensitive to ribosome-targeting inhibition. Our initial observations revealed that ribosome-targeting therapy could regulate T cell and macrophage states. Thus, we first isolated T cells from the abovementioned large-scale human scRNA-seq data (Fig. S2d) and obtained CD4^+^ T cells, CD8^+^ T cells and Tregs according to canonical markers (Fig. S5a–d). Trajectory analysis revealed that the ribosomal state was associated with differentiation progression in CD8^+^ T cells (Fig. S5e). Trajectory-initiated CD8^+^ T cells displayed a naive-like phenotype with an inactive ribosomal state but ended in an active ribosomal state with relatively high cytotoxicity and exhaustion states (Fig. S5f), suggesting that ribosome biogenesis may be correlated with the effectiveness and potential side effects of CD8^+^ T cells. A recent study uncovered unique stress response T cell (T_str_) that beyond the classical T cell distinction [35]. CD8^+^ T_str_ cells are characterized by heat shock genes (such as HSP70, a protein encoded by *HSPA1A/B*) and are associated with immunotherapeutic-resistance [35]. We identified 15 clusters and defined 19 transcriptional states in CD8^+^ T cells refer to this study (Figs. 3a and S5g). CD8^+^ T cells in the active ribosomal state were positively correlated with the stress response state and cell differentiation in tumours (Fig. 3a, b). We confirmed the existence of CD8^+^ T_str_ cells with the active ribosome biogenesis within the TME using clinical samples, which revealed that HSP70^+^ CD8^+^ T cells were relatively enriched in higher rRNA levels compared with HSP70^-^ CD8^+^ T cells (Fig. 3c, d). We also analysed CD4^+^ T cells and observed similar results (Fig. S6a–f).

We then isolated T cells from the abovementioned ribosome-targeting therapy scRNA-seq data (Fig. 3e). CX-5461 treatment promoted CD8^+^ T cell activation and the secretion of granzymes, perforin and cytokine (Fig. S5h). In line with the human data, HSP70^+^ CD8^+^ T cells displayed a more ribosomal state in the model mice (Fig. 3f). Moreover, CX-5461 selectively inhibited cellular ribosome biogenesis of HSP70^+^ CD8^+^ T cells (Fig. 3g), without inducing DNA damage response signaling (Fig. S5i). Impressively, HSP70^+^ CD8^+^ T cells were drastically reduced upon in CX-5461 treatment, underscoring the pivotal role of hyperactivated ribosome activity in sustaining the number of these non-tumour cells within the TME (Fig. 3h). Compared with HSP70^-^ CD8^+^ T cells, HSP70^+^ CD8^+^ T cell presented lower granzyme levels, and CX-5461 treatment could induce HSP70^-^ CD8^+^ T cell-secreted granzymes (Fig. 3i). These findings hint that HSP70^+^ CD8^+^ T cells are sensitive to ribosome inhibition, and as a player in targeting ribosome-mediated T cell state reprogramming. Additionally, Tregs were decreased in CX-5461 treatment samples from the CT26 model (Fig. 3h). Furthermore, the ST data suggested that CD8^+^ T_str_ and Tregs showed more active ribosome biogenesis (Fig. 3j). Clinically, we used the optimal cutoffs for ribosomal state to best stratify patients with *CD8A*^High^ [14]. Notably, although CD8^+^ T cells are generally considered to be mainly anti-tumour immune cells, cases with high intratumoural ribosome biogenesis-active CD8^+^ T cell infiltration have adverse outcomes in numerous cancers (Figs. 3k and S6g).

### Ribosome-targeting therapy-sensitive T cells are essential for immunotherapy efficacy

**Fig. 4:**
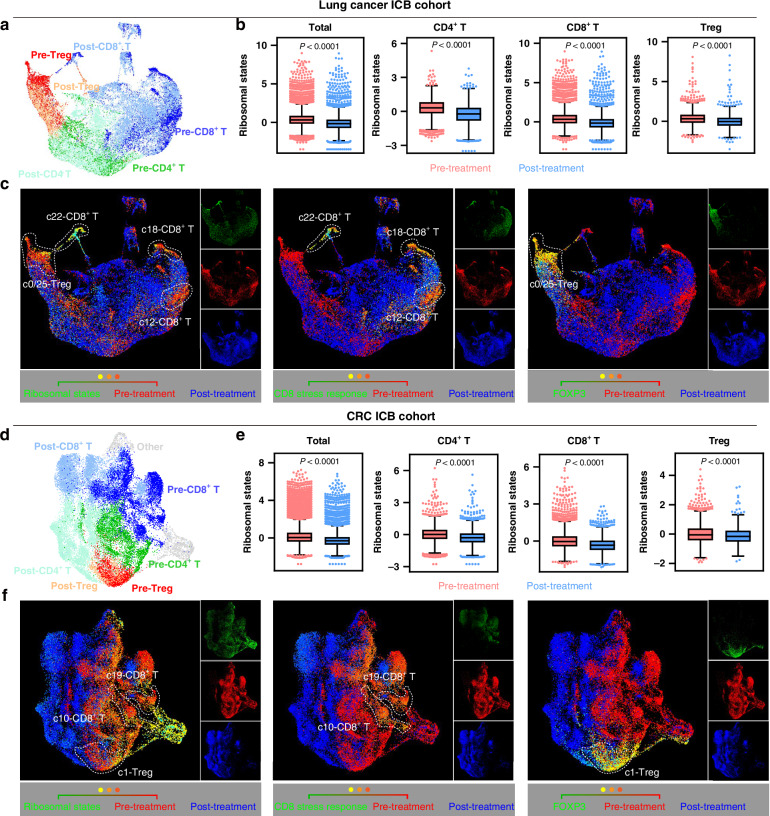
Ribosome-targeting therapy-sensitive T cells are essential to immunotherapy efficacy. **a** UMAP plots showing T cell subtypes grouped by pre- and post-treatment of ICB in cases with lung cancer. **b** Boxplots showing T cells and each subtype cellular ribosome biogenesis grouped by pre- and post-treatment of ICB in cases with lung cancer. **c** Pseudo immunofluorescence based on UMAP plots showing cellular ribosome biogenesis (left), CD8^+^ T cell stress response state (middle) and FOXP3 (right) within T cells marked by pre- and post-treatment of ICB in cases with lung cancer. **d–f** Similar to (**a**–**c**), but in ICB treatment cases with CRC.

Our data suggest that ribosome-targeting therapy regulates T cells, decreasing HSP70^+^ CD8^+^ T (T_str_) cells and Tregs. We therefore aimed to assess the clinical relevance of these T cells using scRNA-seq datasets from previous immunotherapy studies [36, 37] of patients who underwent ICB therapies. T cells from site-matched responsive patients who underwent ICB were isolated, then clustered and annotated (Figs. 4a, d and S7a, b). In both two cohorts, ICB treatment significantly inhibited the global and subtype T cell ribosomal states (Fig. 4b, e). Next, T cells were further unsupervised learned and a series of clusters were identified. The ribosomal state was correlated with the stress response state and pre-treatment biopsies (Fig. S7c, d). Among CD8^+^ T cell clusters, c22, c12 and c18 cells of the lung cancer cohort were the top three with respect to ribosome biogenesis activity (Fig. S7c left), and were located mainly in pre-treatment samples (Fig. 4c left). These clusters/regions featured a stronger stress response CD8^+^ T state (Fig. 4c middle). Likewise, in the CRC cohort, CD8^+^ T cells of c10 and c18 clusters were the top two ribosome biogenesis clusters (Fig. S7d left), and were located mainly in pre-treatment samples (Fig. 4f left), indicating a relatively higher stress response CD8^+^ T states (Fig. 4f middle). For Treg clusters, these cells demonstrated an active ribosomal state and were predominantly located in pre-treatment samples (Fig. 4c, f left and right). Overall, during the ICB treatment of responsive patients, there was a substantial decrease in ribosome-targeting therapy-sensitive T cells, suggesting that these cells are essential for immunotherapy efficacy.

### Immunosuppressive-like TAMs are sensitive to ribosome-targeting therapy

**Fig. 5:**
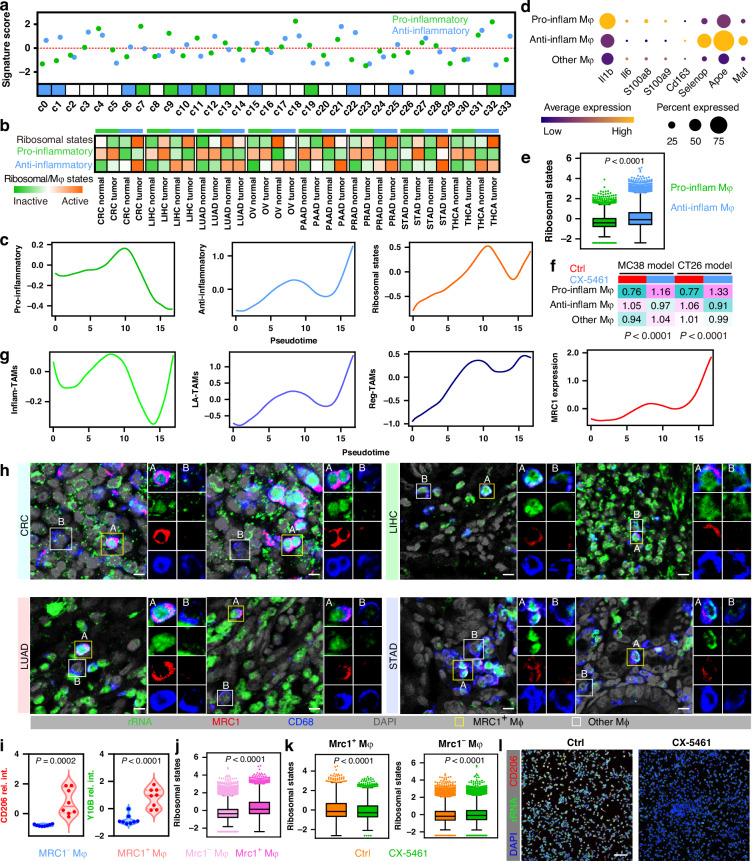
Immunosuppressive-like TAMs are sensitive to ribosome-targeting therapy. **a** Dotplot showing the comparison of pro/anti-inflammatory macrophage signature levels in each cluster (up), indicates pro/anti-inflammatory macrophage definition (bottom). **b** Heatmap showing the cellular ribosome biogenesis and pro/anti-inflammatory states grouped by tumor and tissue types within macrophages (Mφ). **c** Expression pattern of levels of pro/anti-inflammatory states and cellular ribosome biogenesis along pseudotime in the trajectory of macrophages. **d** Dotplot showing expressions of marker genes of pro- and anti-inflammatory macrophages in tumor murine models. Dot size indicates proportion of cells expressing genes, dot color indicates mean expression of genes. **e** Boxplots showing cellular ribosome biogenesis grouped by pro- and anti-inflammatory macrophages of tumor murine models. **f** Heatmaps showing tissue prevalence of pro- and anti-inflammatory macrophages estimated by Ro/e score grouped by CX-5461 pre- and post-treatment samples of tumor murine models. **g** Expression pattern of levels of Inflam/LA/Reg-TAM states and MRC1 along pseudotime in the trajectory of macrophages. **h**, **i** Multicolor-immunostaining of rRNA (Y10B), macrophages (CD68) and anti-inflammatory/LA/Reg-TAM states marker (MRC1) in tissues of CRC, LIHC, LUAD and STAD (**h**). Scale bar for all immunostaining images = 20 µm. ROI A represents MRC1^+^ TAMs, B represents MRC1^-^ TAMs, (**i**) showing the intensity of MRC1 and rRNA levels for each ROI in grouped by MRC1^+^ and MRC1^-^ TAMs. **j** Boxplots showing cellular ribosome biogenesis grouped by Mrc1^+^ Mφ and Mrc1^-^ Mφ of tumor murine models. **k** Boxplots showing cellular ribosome biogenesis in Mrc1^+^ Mφ and Mrc1^-^ Mφ of tumor murine models grouped by CX-5461 pre- and post-treatment samples. **l** Immunostaining of anti-inflammatory/LA/Reg-TAM states marker CD206 (red) and rRNA (Y10B, green) in TAMs upon CX-5461 treatment. Immunosuppressive-like TAMs obtained from conditioned medium treated RAW264.7 cells. Scale bar =100 µm.

TAMs are among the most abundant non-tumour cells in the TME, and are widely referred to as pro-inflammatory (M1-like) or anti-inflammatory (M2-like) cells to describe their diversity [18]. Thus, we firstly isolated macrophages and defined them as pro/anti-inflammatory macrophages referring to signatures from a previous study (Fig. 5a) [38]. Overall, the ribosomal state of anti-inflammatory TAMs was higher than pro-inflammatory TAMs and upregulated in tumour tissues (Fig. 5b). Interestingly, in CRC, the ribosomal state of pro-inflammatory TAMs was downregulated in tumours. Trajectory analysis showed that the ribosomal state was correlated with differentiation progression, which is accompanied by overall decreased pro-inflammatory and increased anti-inflammatory states in tumours (Figs. 5c and S8a). These results suggest that TAM cellular ribosome biogenesis levels are correlated with transcriptional reprogramming from the pro- to the anti-inflammatory differentiation axis in tumourigenesis. We further isolated and annotated macrophages from the abovementioned scRNA-seq data of ribosome-targeting therapies (Fig. 1b and 5d). In line with the human data, anti-inflammatory TAMs displayed a more active ribosomal state in mouse models (Fig. 5e), and a decrease of these cells in CX-5461 post-treatment samples emerged, while pro-inflammatory TAMs were increased (Fig. 5f). These results suggest that anti-inflammatory TAMs are more sensitive to ribosome inhibition.

The nomenclature of M1/M2 albeit widely used to describe the in vitro activation state of macrophages, remains limited in vivo. We have uncovered that CX-5461 treatment inhibited representative markers of LA/Reg-TAMs in macrophages from model mice (Fig. 1g). Trajectory analysis of human scRNA-seq data showed that the ribosomal state and differentiation progression were correlated with elevated LA/Reg-TAMs, while the Inflam-TAM signature level decreased (Figs. 5c, g and S8a, b). We noted that CD206 (*MRC1* encodes protein) is expressed in human/mouse LA/Reg-TAMs at both mRNA and protein levels [18]. We thereby selected CD206 to label LA/Reg-TAMs in further assays, and observed that the ribosomal state and differentiation progression was also correlated with *MRC1* levels (Fig. 5c, g). Clinical sample validation demonstrated that *MRC1*^+^ macrophages showed relatively higher rRNA levels than other macrophages did in tumours (Fig. 5h, i). Moreover, scRNA-seq data of ribosome-targeting therapies showed that *Mrc1*^+^ macrophages featured a more active ribosomal state (Fig. 5j). CX-5461 significantly decreased the degree of cellular ribosome biogenesis in *Mrc1*^+^ macrophages (Fig. 5k), but did not induce a DNA damage response (Fig. S8c). Immunostaining analyses were further confirmed that CX-5461 treatment strongly decreased *MRC1*^+^ macrophage proportion and rRNA levels in immunosuppressive-like TAMs (Fig. 5l), but had almost no effect on DNA damage (Fig. S8d). Accordingly, immunosuppressive-like TAMs are more sensitive to ribosome blockade. Clinically, we used the optimal cutoffs for the ribosomal state to best stratify patients with *CD68*^High^ [14]. Cases with high intratumoural ribosome biogenesis-active macrophage infiltration showed adverse outcomes in multiple cancers (Fig. S8e, f).

### Targeting ribosomes in the TME promotes ICB efficacy

**Fig. 6:**
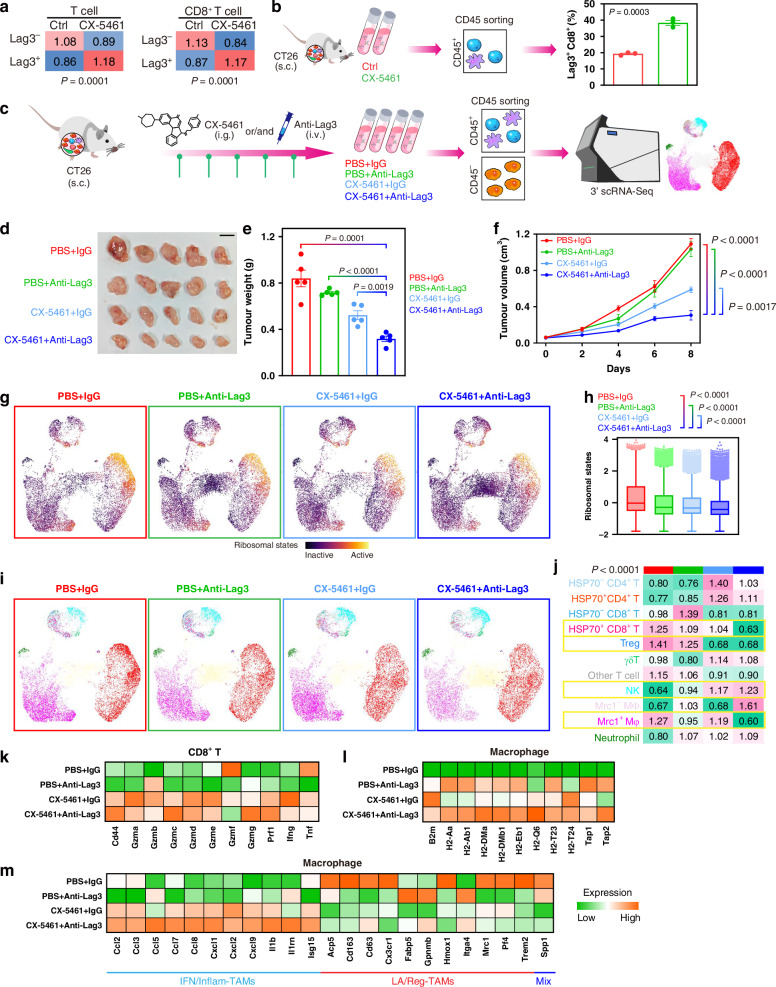
Targeting ribosome in the TME promotes ICB efficacy in the CT26 murine model. **a** Heatmaps showing tissue prevalence of Lag3^+^ and Lag3^-^ T/CD8^+^ T cells estimated by Ro/e score grouped by ctrl and CX-5461 treatment. **b** Bar plots showing proportion of Lag3^+^ CD8^+^ T cells estimated by flow cytometry assays grouped by ctrl and CX-5461 treatment. **c** Schematic showing therapy experiments of combined impact of CX-5461 and anti-Lag3 therapy. **d** Image of tumors at the end point. Scale bar = 1 cm. **e** Tumor weight at the end point. Data are shown as means ± SEM. **f** Tumor size over time during therapy. Data are shown as means ± SEM. **g** UMAP plots showing cellular ribosome biogenesis from each treatment group. **h** Boxplots showing cellular ribosome biogenesis from each treatment group. **i** UMAP plots showing cell types across treatment groups. **j** Heatmap showing tissue prevalence of each CD45^+^ cell type estimated by Ro/e score across treatment groups. **k** Heatmap showing expressions of activation and cytotoxic granules in CD8^+^ T cells across treatment groups. **l** Heatmap showing expressions of antigen processing and presentation genes in macrophage across treatment groups. **m** Heatmap showing expressions of INF/Inflam/LA/Reg-TAMs markers in macrophage across treatment groups.

ICs promote immune evasion and regulate anti-tumour immune responses within the TME. Immunologically cold tumours rarely respond to ICB therapies. Our abovementioned findings demonstrate that ribosome-targeting inhibition strongly reshapes the protumourigenic TME to anti-tumour TME, including the CT26 model, which is a microsatellite stability murine model that relatively resistant to ICB [39, 40]. Thus, we analysed ICs referred to previous studies [12, 41] in the CT26 model with ribosome-targeting therapy. CX-5461 treatment induced most ICs in cancer cells, T/CD8^+^ T cells and macrophages, respectively (Fig. S9a–d). A previous study exhibits that CX-5461 combined with anti-PD-L1 (*CD274*) enhances therapeutic efficacy in CRC [11]. Interestingly, *Cd274* was upregulated in T/CD8^+^ T cells and macrophages after CX-5461 treatment (Fig. S9b–d). We noted that Lag3, the third IC that is a promising immunotherapeutic target [42], was more highly expressed than *Cd274* in T/CD8^+^ T cells, and was upregulated in post-treatment samples (Figs. S9b, c and 6a). Flow cytometry of single-cell suspensions from tumour tissues further hints that CX-5461 administration induced *Lag3* in CD8^+^ T cells (Fig. 6b).

We finally investigated whether targeting ribosomes could enhance ICB efficacy in the immunotherapeutic-resistance tumours by testing the combined impact of CX-5461 and anti-Lag3 in the CT26 model (Fig. 6c). CX-5461 modestly inhibited tumour growth and significantly sensitized tumours to anti-Lag3 immunotherapy (Fig. 6d–f), but had no adverse effects on body weight (Fig. S10a). The combination therapy most significantly reduced tumour size and weight (Fig. 6e, f). ScRNA-seq was used to further explore how CX-5461 in conjunction with anti-Lag3 therapy treatment influences the TME (S10b–d). Impressively, the combination treatment further decreased ribosomal states (Fig. 6g, h) and a series of ribosome-targeting therapy-sensitive cell subtypes, including HSP70^+^ CD8^+^ T cells, Tregs and *Mrc1*^+^ macrophages (Fig. 6i, j). Of note, combination treatment elevated CD8^+^ T cell activation states, and improved the expression of CD8^+^ T cell-secreted granzymes, perforin, IFN-γ and TNF-α (Fig. 6k). Combination administration also increased the proportion of NK cells (Fig. 6j) and their granzymes and perforin secretion (Fig. S10e). Moreover, macrophages presented increased levels of antigen processing and presentation capacity (Fig. 6l), and increased lFN/Inflam-TAM marker levels, while decreased LA/Reg-TAM marker levels in combination treatment samples (Fig. 6m). Taken together, our findings indicate that ribosome-targeting therapy remodels the TME, remarkably enhancing the anti-tumour effects of Lag3 blockade in immunologically cold tumours.

## Discussion

Hyperactive ribosome biogenesis in cancer cells is a hallmark of tumours. We have explored ribosome-related genes predominantly in cancer cells at the bulk level [5, 6]. In the present study, we functionally characterized and systematically probed how ribosome-targeting therapy alters the TME ecosystem. Based on our findings, we propose that targeting ribosomes may represent a therapeutic entry point to enhance ICB efficacy in immunotherapeutic-insensitive tumours.

CX-5461, a ribosome-targeting inhibitor, has been investigated in phase I and II trials, which have focused mainly on patients with *BRCA1/2* deficient tumours [7–10]. To date, biomedical and mechanical studies on CX-5461 in tumours have focused predominantly on cancer cells themselves. Indeed, ribosomes can heterogeneously regulate both cancer and non-cancer cell fates within the TME. Despite these early findings, there remains a critical need for an improved understanding of the TME alternations mediated by CX-5461 based on immunocompetent pre-clinical models and the TME atlas to guide the effective use of this therapy. Here, we firstly generated the single-cell TME landscape of CX-5461-treated tumour pre-clinical models, and observed that CX-5461 treatment induced CD8^+^ T and NK cell-secreted cytotoxic granules. Moreover, CX-5461 treatment enhanced macrophage pro-inflammation reprogramming. Therefore, ribosome-targeting therapy may reform the tumour permissive microenvironment, eliciting anti-tumour immune responses.

As ribosomes can translationally control both cancer and non-cancer cells, the deconvolution of bulk expression data may hard to provide an accurate estimate of the cellular ribosome biogenesis level in given samples. We therefore constructed NMF-programs consistent with ribosome-related signature levels in tumour epithelial cells and defined a 73-metagene ribosomal state, aiding in the identification of cell subtypes that are sensitive to ribosome-targeting therapy. These metagenes contain several canonical ribosome-related genes, such as *NPM1/3*, *EEF1E1* and *POLR3K*. One of these genes, *IMPDH2*, is a rate-limiting enzyme for de novo guanine nucleotide biosynthesis and promotes tumourigenesis by regulating GTP metabolic reprogramming [43]. Interestingly, this rate-limiting enzyme is also associated with increased rRNA/tRNA synthesis in glioblastoma [44]. We noted that canonical RP genes are not included in this signature. This finding is not unexpected because metagenes tend to reflect the relevant phenotype of the target biological process/pathway according to expression patterns, rather than including key genes that are directly involved in this process/pathway. For example, serial studies have identified signatures that reflect hypoxia status and a 15-metagene signature appears to perform the best [45, 46]. However, as a master regulator of the cellular and systemic homeostatic response to hypoxia, *HIF1A* is not included in metagenes. Importantly, our ribosomal state metagenes are not be disrupted by filtering (mitochondrial) ribosomal L/S-protein genes when single-cell data quality control is needed. For instance, official CRC Visium HD data lack information on RP genes, probably due to the detection technology or quality control of filtering ribosomal L/S-protein genes. Therefore, RP genes cannot be used to measure ribosome biogenesis activity in this data. Additionally, it is generally difficult to measure ribosome biogenesis directly in a large number of clinical samples for cancer diagnosis and hardly implemented. Hence, it is necessary to identify clinical transcriptome signatures reflecting ribosome biogenesis, such as ribosomal states in this study.

Our findings hint that active ribosomal states are associated with tumourigenesis and poor clinical outcomes. Remarkably, we uncovered a series of immune cells, including HSP70^+^ CD8^+^ T (T_str_) cells, Tregs and *MRC1*^+^ (immunosuppressive-like) TAMs that decreased after CX-5461 administration, suggesting that these cells may be sensitive to ribosome-targeting therapy. Cancer cells exploit cellular stress response pathways to enhance their tolerance. Ribosomes are selectively recruited to complete protein synthesis when various stresses occur [47]. A well-designed study identified unique T cells with a stress response state (T_str_) marked by HSP70 [35]. Interestingly, an early study in the 1990s suggested that HSP70 contributes to ribosome biogenesis [48]. Further studies have shown that HSP70 promotes ribosome biogenesis and protein synthesis during acute stress, and mediates the incorporation of ‘orphan’ RPs constituents into functional ribosomes when cells recover from heat shock [49, 50]. Thus, co-existence of HSP70 and high ribosome biogenesis may explain why HSP70^+^ CD8^+^ T (T_str_) cells are sensitive to ribosome-targeting therapy: the protumourigenic and immunosuppressive microenvironment during tumourigenesis requires these T cells, enhancing their cellular ribosome biogenesis by HSP70 regulation during differentiation. Some patients treated with anti-PD-1 antibodies experience rapid cancer progression, referred to as hyperprogressive disease. PD-1 blockade augmented the proliferation and immunosuppression of Tregs [51]. Thus, depletion of Tregs via ribosome-targeting strategy and combined with an anti-PD-1 antibody may help treat and prevent hyperprogressive disease. Furthermore, ribosome-targeting therapy-sensitive T cells were markedly downregulated in ICB post-treatment samples from responsive patients, suggesting potential ribosome biogenesis-related resistance mechanisms to ICB therapies [35]. Hence, ribosome-targeting inhibition combined with ICB could be an interesting avenue for future tumour immunotherapies. These results also suggest a plasticity of cellular ribosome biogenesis within the protumourigenic and immunosuppressive microenvironment. Intriguingly, a recent study in supercentenarians (SCs) revealed that, as the most prominent molecular hallmark at the single-cell level, immune cell types with high ribosome levels were associated with a low inflammatory state and slow ageing of SCs [52]. This observation indicated that cells in high ribosome states at the single-cell level in the SC microenvironment could prevent inflammation and ageing. Thus, one of the explanations for this and our findings is that ribosomal status plasticity of the microenvironment would serve different biomedical themes, like the philosophical assertation “where you stand depends on where you sit”. This plasticity once co-opted by tumours, promotes uncontrolled cancer cell proliferation and sustains immunosuppressive non-tumour cell numbers, fueling the evolution of the tumourigenesis.

Interestingly, CX-5461 treatment induced partial IC expression in the ICB-insensitive model. For instance, *Cd274* is upregulated in macrophages of post-treatment samples. This could be due to the increased proportion of IFN-TAMs, which are associated with high expression of *CD274* though resemble M1-like macrophages [18]. Our single-cell atlas demonstrated that CX-5461 treatment remodeled the TME, inhibited immunotherapeutic-resistance T cells, reinforced anti-tumour T/NK cells cytotoxicity factor levels and macrophage pro-inflammation capacity, and induced IC expressions. These features that occur in response to CX-5461 administration seem similar to immune overdrive TME phenotypes reported in previous studies, which may exhibit better responses after ICB [12, 14, 53]. Patients with high microsatellite instability cases could benefit from immunotherapy. An explanation is that these cancer cells probably harbor many somatic mutations that encode potential neoantigens, and are therefore likely to be immunogenic, as well as to induce ICs upregulation [19]. Interestingly, our initial evidence suggests that CX-5461 leads to the production of misfolded proteins that can be displayed on the surface of cancer cells, most likely as unique neoantigens. In line with this, a recent study demonstrated that translation dysregulation could be a source for targetable antigens in cancers [54]. We also demonstrated that CX-5461 could directly decrease ribosome biogenesis in immunosuppressive-like TAMs. Hence, both cancer cell-dependent and -independent mechanisms likely contribute to TME reprogramming under global ribosome biogenesis blockade. In some patients, tumour-intrinsic immunosuppression can lead to a cold tumour [55]. A recent research exhibited that CX-5461 combined with anti-PD-1/PD-L1 therapy probably turns cold tumours into hot [11]. We found that CX-5461 significantly sensitized CT26 tumours to anti-Lag3 immunotherapy, leading to potent tumour regression. Extensive single-cell transcriptomic analysis revealed that combined therapy yielded deeper anti-tumour immune responses. Our findings unveil that ribosome biogenesis blockade, to some extent, could reinstate immunosurveillance and provide novel strategies to enhance ICB efficacy in cold tumours.

Overall, this study is the first to provide a single-cell atlas and potential mechanistic insight into ribosome-targeting therapy for reshaping the TME and enhancing ICB efficacy in pre-clinical models. Given the substantially improved anti-tumour immune responses and anti-Lag3 efficacy following CX-5461 treatment, dual blockading of ribosome biogenesis and LAG3 may be efficacious in immunotherapeutic-resistance cases and is considered a beneficial therapeutic approach. Future investigations to explore the TME reprogramming efficacy of the ribosome-targeting therapy in more models and non-epithelial (-like) solid cancer types, such as glioma, are necessary. In addition to CX-5461, other ribosome-targeting inhibitors, such as Rbins (Ribozinoindoles) [56], which are potent, reversible and specific chemical inhibitors of eukaryotic ribosome biogenesis, could be used in future studies.

## Materials and methods

### Cell culture and animal resources

Mouse CRC cell lines MC38 and CT26, mouse monocytic/macrophage cell line RAW264.7 were obtained from the Mcellbank (Cat# M-C2027, M-C2088 and M-C2017). Human CRC cell line RKO, breast cancer cell line MCF7 and renal cancer cell line A498 were obtained from the American Type Culture Collection (ATCC, Cat# CRL-2577; HTB-22; HTB-44) and were free of mycoplasma contamination. MC38 was cultured in RPMI (Eallbio, Cat# 03.4007c) medium containing 10% FBS (Sigma, Cat# F8687-500ML). CT26 was cultured in RPMI medium containing 1% Penicillin-Streptomycin (Beyotime, Cat# C0222). RKO and MCF7 were grown in DMEM (Eallbio, Cat# 03.1006c) medium suppled with 10% FBS and 1% Penicillin-Streptomycin. A498 were incubated in MEM (HyClone, Cat# SH30024.01) medium supplemented with 10% FBS and 1% Penicillin-Streptomycin. All these cells were incubated at conditions of 37°C in humidified 5% CO_2_ atmosphere. C57BL/6 and BALB/c mice (5 weeks, female), which were used for the animal research, were purchased from Zhuhai Bestest Biotechnology Co., Ltd. (Zhuhai, China). All animal experiments were reviewed and approved by the Institutional Animal Care and Use Committee of Shenzhen Institutes of Advanced Technology, Chinese Academy of Science.

### Ribosome-targeting therapy experiment

MC38 and CT26 cells were collected and subjected to re-suspension by sterile PBS (Boster, Cat# pyg14190) after washing three times using sterile PBS. MC38 or CT26 cells (6 × 10^5^ cells resuspended in 200 mL PBS) were subcutaneously injected into the right armpit of the forelimb of BALB/c mice. Seven days after tumour inoculation, five tumour-bearing mice received CX-5461 (MCE, Cat# HY-13323) treatment at a dose of 50 mg/kg [57] via gavage every two days (five times in total), and another five tumour-bearing mice were used to control tests through oral administration of equal volume PBS. All of the test mice were sacrificed and the tumour tissues were harvested for further scRNA-seq analysis.

### Ribosome-targeting inhibition and anti-Lag3 combination therapy experiment

The model of CT26 subcutaneous transplant tumours were established in line with aforementioned approaches. Then, these tumour-bearing mice were divided randomly among four groups (*n* = 5): PBS/IgG, PBS/anti-Lag3 (Bioxcell, Cat# BP0174), CX-5461/IgG (Bioxcell, Cat# BP0088), CX-5461/anti-Lag3. The administration method is following: CX-5461 (50 mg/kg) [57] through gavage, antibody (200 μg/mouse) via tail vein. These mice were treated once per two days in total five times. The volumes of tumours and body weights of the mice were measured, every two days, and the calculation was conducted with the formula volume: V (mm^3^) = length × wide × wide/2. Fourteen days after the first injection, these mice were humanely sacrificed and their tumour tissues were excised for further scRNA-seq analysis.

### ScRNA-seq and analysis

Tissues from abovementioned therapy experiments were performed by experimental personnel in the laboratory of SeekGene Biotechnology Co., Ltd. (Beijing, China). Single cell suspensions from each sample were sorted using CD45 MicroBeads (Miltenyi, Cat# 130-052-301) and then integrated cells following CD45^+^:CD45^-^ = 2:1 (CX-5461) /3:1 (CX-5461/anti-Lag3). The scRNA-seq libraries were prepared using the SeekOne® Digital Droplet (DD) Single Cell 3′ library preparation kit (SeekGene, Cat# No. K00202) and related platform following the manufacturer protocol and libraries sequencing was performed in an Illumina NovaSeq 6000. SeekSoul® Tools was used to proceed raw data and generated Unique Molecular Identifier (UMI) count matrices. R package Seurat 4 was used to further processed and annotated scRNA-seq UMI count matrices data [58]. Cells with fewer than 200 detected genes were removed and reserved cells was filtered based on total UMI count detected per cell (nCount RNA), number of genes detected per cell (nFeature RNA), log_10_(nFeature RNA)/log_10_(nCount RNA) per cell and ratio of mitochondrial genes’ UMI counts per cell. Then, filtered cells were integrated and UMI counts were normalized with standard library size scaling and log transformation. We applied the *FindVariableFeatures* function to detect highly variable genes (HVGs) for normalized matrix. *ScaleData*, *RunPCA* and *RunUMAP* functions were applied sequentially with parameter features set to HVGs. R package DoubletFinder was used to identifies and removed doublets formed from transcriptionally distinct cells [59]. Ro/e approach was used to quantification of tissue enrichment of each cell type according to previously description [38]. Ro/e values over than one indicates enrichment, whereas Ro/e values less than one indicates depletion of cells in tissue.

### Flow cytometry

Single-cell suspensions from tumour tissues treated with CX-5461 were harvested using Tumour Tissue Mononuclear Cell Separation Solution (Solarbio, P4740, China). After filtered through 70 μm cell strainer, they were blocked with Rat Anti-Mouse CD16/CD32 (1ug per 1×106 cells, BD, 553141) at 4°C for 10 min. Subsequently, these cell suspensions were stained with FVS440UV (1:1000, BD, 566332), Alexa Fluor® 700-CD45, FITC-CD3, PE-CD8a, APC-LAG3, and then analyzed by BD LSRFortessa flow cytometry (BD, USA).

### Clinical samples curation

In-house clinical samples selected for multicolor-immunostaining were provided by the Department of Pathology, Jiangnan University Medical Center following standard operating procedures. This study was approved by the Medical Ethic Committee of Jiangnan University Medical Center. All patients provided written informed consent.

### Multicolor-immunostaining

Multicolor-immunostaining of paraffin-embedded (FFPE) tissues were performed using PANO 7-plex IHC kit (Panovue, Cat# 10216100100) according to manufacturer’s instruction. Briefly, FFPE tissue slides were first deparaffinized and then incubated sequentially with primary antibodies EpCAM (Santa, Cat# sc-25308), CD45 (PTG, Cat# 60287-1-Ig), Anti-ribosomal RNA (Abcam, Cat# ab171119), CD8A (GZLBP, Cat# IR024), CD4 (Abcam, Cat# ab40763), HSP70 (PTG, Cat# 10995-1-AP), CD68 (CST, Cat# #26042) and CD206 (PTG, Cat# 60143-1-Ig) were respectively followed by horseradish peroxidase-conjugated secondary antibody incubation and tyramide signal amplification. The slides were microwave heat-treated after each tyramide signal amplification operation. Nuclei were stained with DAPI after all the antigens above had been labeled. The immunostaining images were captured via microscopy (Carl Zeiss), which captures the fluorescent spectra at 20-nm wavelength intervals from 420 to 720 nm with identical exposure time. For each slide, two or four fields of immune cell enriched tumoural area were selected for image capture. All scans for each slide were then superimposed to obtain a single image. Multilayer images were imported to the ImageJ software for quantitative intensity analysis.

### In vitro immunostaining

CT26 cells (1 × 10^5^ cells per well) were seeded on coverslips in 24-well plates and cultured overnight. After attachment, the cells were treated with 200 nM CX-5461 for 24 h, and PBS or 4 μg/mL 5-FU served as negative or DNA damage positive control. In vitro immunosuppressive-like TAMs were induced by CT26 cell free supernatant according to our descriptions [60]. Subsequently, for the DNA damage assay, the immunofluorescence of γ-H2AX was performed referred to our previous methods [6, 61], and the γ-H2AX (HUABIO, Cat#ET1602-2) or CD206 (HUABIO, Cat#ET1702-04) antibodies were diluted at a ratio of 1:2000 or 1:200; the rRNA assay was performed as our previous reports [6]; for the misfolded proteins detection, CX-5461-treated CT26 cells were evaluated using PROTEOSTAT aggresome detection assay kit (Enzo Life Sciences, ENZ-51035) as previously described [62]. F-actin stained with iFluor™ 647 phalloidin (Yeasen, Cat#40762ES75) served as intracellular location. After incubated with secondary antibody and DAPI, these cells were imaged by confocal laser scanning microscope (Carl Zeiss).

### Polysome profiling assay

To block translational elongation, 1% volume of 100x cycloheximide (Acmec, Shanghai, China) was applied to cell culture medium. Next, cells were incubated under the original conditions for 15 min. Then cells were washed by pre-chilled PBS buffer. Subsequently, cell samples were performed by experimental personnel in the laboratory of CHI BIOTECH Co., Ltd. (Shenzhen, China) refer to the previous study [63].

### Public data and resources

ScRNA-seq data: Only 10X Genomics 3’ scRNA-seq data were used in integrated Pan-cancer scRNA-seq dataset for avoiding comparing data collected from different technologies. About Pan-cancer single-cell dataset, seven cancer types were obtained from GEO (http://www.ncbi.nlm.nih.gov/geo), including CRC (GSE132465), LIHC (GSE149614), OV (GSE184880), PAAD (GSE212966), PRAD (GSE193337), STAD (GSE163558) and THCA (GSE184362); LUAD (https://codeocean.com/capsule/8321305/tree/v1). Due to large-scale cells of different samples, we used the R package simspec after *RunPCA* functions applying to integrate these cells across cancer types, samples and batches, based on unsupervised reference-free data representation, cluster similarity spectrum (CSS) algorithm [64]. After improving data integration and removing batch effects, 489706 cells encompassing eight cancer types and 116 samples were presented and established a Pan-cancer single-cell dataset. ScRNA-seq datasets of ICB therapy were available from GSE179994 and GSE236581.

Bulk transcriptional level data: TCGA Pan-cancer datasets were downloaded from the Genomic Data Commons Data Portal (https://portal.gdc.cancer.gov/). TCGA tumour samples with gender, race, stage, histological type and survival time information were used in clinical analysis. AOM/DSS-induced CRC tumourigenesis in vivo datasets were available from GSE44904 and GSE178145. Datasets of ribosome biogenesis dysregulated using pharmacologic and biochemical approaches were obtained from GSE197487, GSE213472, GSE212714, GSE214652 and GSE201899. Cancer cell expression data was obtained from Depmap (https://depmap.org/portal/).

Other: About ST dataset, CRC spatial HD data was obtained from https://www.10xgenomics.com/datasets; CRC spatial data was obtained from http://www.cancerdiversity.asia/scCRLM/, OV and BRCA spatial data was obtained from GSE211956 and GSE210616, respectively. Cancer cell drug sensitivity dataset was obtained from Depmap (https://depmap.org/portal/). ST dataset count matrices were processed using R package Seurat 4 [58]. We used spatial position code and extracted expression of *EPCAM*, *DCN* and *PTPRC* (CD45 mRNA symbol) to display epithelial cells, immune cells and fibroblasts, respectively.

### Signature level evaluation

Signature levels scoring in single-cell and ST datasets using the R package UCell [65]. This algorithm based on the Mann-Whitney U statistic, depend only on the relative gene expression in individual cell, therefore not affected by dataset composition. Ribosome-related signatures and Hallmark gene sets were obtained from molecular signatures database (MSigDB, https://www.gsea-msigdb.org/gsea/msigdb/collections.jsp). Transcriptional states gene set were available on the previous analysis [35]. Transcriptomic diversity gene sets of TAMs were collected from the previous study [18]. Signature bulk-level calculation was used *ssGSEA* algorithm of R package GSVA [66].

### Identifying ribosome-related gene expression programs

Gene expression programs are NMF cell clusters defined by common tasks, such as ribosome-related biological processes in this study. NMF approach was utilized to characterize ribosome-related programs in tumour epithelial cells and 59 tumour samples contains more than 200 epithelial cells were used. For each tumour, programs were learned by NMF algorithm and generated related NMF program score. The top nine ranked (set rank = 2: 10) co-expressed gene programs in each tumour sample were dissected. How to selected the NMF program in each sample? When set rank = *n* (*n* = 2 ~ 10), we obtain P1, P2, …… P*n* programs, total (2 + *n*) × *n*/ 2 programs in each tumour; we correlated each program’ score with canonical five ribosome signature scores within cells and obtain mean correlation value of each program; thus, the program that showed most positive correlation level in each tumour as mean-maxed programs, also obey two criteria: (1) at least two programs’ cell count over 10% in candidate rank of its tumours; (2) mean correlation value of program over 0.1. For instance, in CRC SMC20-T sample, programs P1, P3 and P4 count over 10%; mean correlation value of P4 program was max and over 0.1 (R_mean_ = 0.561), therefore P4 was selected. Overall, 348 NMF programs were identified, contains 158 programs that cell count less than 10%. Subsequently, hierarchically clustering of mean correlation value across these programs identified 37 mean-maxed programs were clustered together no matter if programs that cell count less than 10% including. These 37 mean-maxed programs were defined as ribosome-related programs.

### Clinical analysis

Overall survival was evaluated by Kaplan–Meier survival analysis and log-rank test according to our previously described methods [5, 12, 14]. For two-group survival, samples were divided into high and low groups based on the expression level of ribosomal states, the lowest log-rank *P* value was select from the 10th to 90th percentiles of the samples. For three-group survival, the mean expression value of *CD8A* or *CD68* was used to divide the total cases into the *CD8A*/*CD68*^High^ and CD8A/CD68^Low^ groups, then used the optimal cutoffs for ribosomal states to best stratify *CD8A*/*CD68*^High^ cases into two groups based on abovementioned of two-group survival analysis. About multivariate Cox regression model analysis, no collinearity was observed among these factors in each cancer type we displayed with tolerance >0.1 and VIF < 10.

### iCMS analysis

Metagenes of iCMS2-3 up/down were obtained from the previous study [33]. Then, we calculated iCMS2-3 up/down scores using R package UCell and scaled it [65]. iCMS2 subtype clusters were defined as either of iCMS2 up and iCMS3 down scores over than either of iCMS3 up and iCMS2 down; iCMS3 subtype clusters were defined as either of iCMS3 up and iCMS2 down scores over than either of iCMS2 up and iCMS3 down. Finally, iCMS2 cells showed iCMS2 up/iCMS3 down scores were over zero and iCMS3 up/iCMS2 down less than zero, while iCMS3 cells showed iCMS3 up/iCMS2 down scores were over zero and iCMS2 up/iCMS3 down less than zero.

### Trajectory analysis

The R package Monocle 3 was used to reconstruct the cellular differentiation trajectory CD8^+^ T, CD4^+^ T and macrophages in tumour samples [35, 67]. Cells were processed and remove batch effects using *preprocess_cds* and *align_cds* functions, respectively. After reducing dimension and clustering cells using *reduce_dimension* and *cluster_cells* functions, a principal graph was displayed on the UMAP as ‘skeleton lines’ based on the *learn_graph* function, indicating the differentiation trajectories. Finally, selected root for the trajectory based on previous knowledge and the *order_cells* function was used to generate pseudotime data.

### Statistical analysis, code availability and visualization

Statistical analyses were performed using GraphPad Prism software 10 and R software 4.1.0. Significance was determined by Mann-Whitney U test, Student’s t test, Kruskal–Wallis test, Chi-squared test, Pearson Correlation Coefficient and log rank Mantel-Cox test, *P* values < 0.05 is considered statistically significant. Detailed information of package used in R software is described in related section of Materials and Methods. Figures were designed, analyzed, and visualized by GraphPad Prism10, R software 4.1.0. and Image J 1.52p.

## Supplementary information

Supplementary Information

## Data Availability

Available of public datasets are described in the ‘Public data and resources’ section. All other data supporting the findings of this study are available from the corresponding author on reasonable request.

## References

[1] Pelletier J, Thomas G, Volarevic S. Ribosome biogenesis in cancer: new players and therapeutic avenues. Nat Rev Cancer. 2018;18:51–63. 29192214 10.1038/nrc.2017.104

[2] Hurt E, Cheng J, Babetaler J, Iwasa J, Beckmann R. SnapShot: Eukaryotic ribosome biogenesis I. Cell. 2023;186:2282.e2281. 37172570 10.1016/j.cell.2023.04.030

[3] Prakash V, Carson BB, Feenstra JM, Dass RA, Sekyrova P, Hoshino A, et al. Ribosome biogenesis during cell cycle arrest fuels EMT in development and disease. Nat Commun. 2019;10:2110. 31068593 10.1038/s41467-019-10100-8 PMC6506521

[4] Fabbri L, Chakraborty A, Robert C, Vagner S. The plasticity of mRNA translation during cancer progression and therapy resistance. Nat Rev Cancer. 2021;21:558–77. 34341537 10.1038/s41568-021-00380-y

[5] Cui K, Liu C, Li X, Zhang Q, Li Y. Comprehensive characterization of the rRNA metabolism-related genes in human cancer. Oncogene. 2020;39:786–800. 31548613 10.1038/s41388-019-1026-9

[6] Cui K, Gong L, Zhang H, Chen Y, Liu B, Gong Z, et al. EXOSC8 promotes colorectal cancer tumorigenesis via regulating ribosome biogenesis-related processes. Oncogene. 2022;41:5397–410. 36348012 10.1038/s41388-022-02530-4

[7] Kusnadi EP, Trigos AS, Cullinane C, Goode DL, Larsson O, Devlin JR, et al. Reprogrammed mRNA translation drives resistance to therapeutic targeting of ribosome biogenesis. EMBO J. 2020;39:e105111. 32945574 10.15252/embj.2020105111 PMC7604608

[8] Bywater MJ, Poortinga G, Sanij E, Hein N, Peck A, Cullinane C, et al. Inhibition of RNA polymerase I as a therapeutic strategy to promote cancer-specific activation of p53. Cancer Cell. 2012;22:51–65. 22789538 10.1016/j.ccr.2012.05.019 PMC3749732

[9] Sanij E, Hannan KM, Xuan J, Yan S, Ahern JE, Trigos AS, et al. CX-5461 activates the DNA damage response and demonstrates therapeutic efficacy in high-grade serous ovarian cancer. Nat Commun. 2020;11:2641. 32457376 10.1038/s41467-020-16393-4 PMC7251123

[10] Khot A, Brajanovski N, Cameron DP, Hein N, Maclachlan KH, Sanij E, et al. First-in-Human RNA Polymerase I Transcription Inhibitor CX-5461 in patients with advanced hematologic cancers: results of a phase i dose-escalation study. Cancer Discov. 2019;9:1036–49. 31092402 10.1158/2159-8290.CD-18-1455

[11] Chung SY, Chang YC, Hsu DS, Hung YC, Lu ML, Hung YP, et al. A G-quadruplex stabilizer, CX-5461 combined with two immune checkpoint inhibitors enhances in vivo therapeutic efficacy by increasing PD-L1 expression in colorectal cancer. Neoplasia. 2023;35:100856. 36442297 10.1016/j.neo.2022.100856 PMC9709093

[12] Cui K, Yao S, Liu B, Sun S, Gong L, Li Q, et al. A novel high-risk subpopulation identified by CTSL and ZBTB7B in gastric cancer. Br J Cancer. 2022;127:1450–60. 35941174 10.1038/s41416-022-01936-x PMC9553888

[13] Liu B, Li S, Cheng Y, Song P, Xu M, Li Z, et al. Distinctive multicellular immunosuppressive hubs confer different intervention strategies for left- and right-sided colon cancers. Cell Rep. Med. 2024;5:101589. 38806057 10.1016/j.xcrm.2024.101589 PMC11228667

[14] Cui K, Yao S, Zhang H, Zhou M, Liu B, Cao Y, et al. Identification of an immune overdrive high-risk subpopulation with aberrant expression of FOXP3 and CTLA4 in colorectal cancer. Oncogene. 2021;40:2130–45. 33627780 10.1038/s41388-021-01677-w

[15] Li R, Ferdinand JR, Loudon KW, Bowyer GS, Laidlaw S, Muyas F, et al. Mapping single-cell transcriptomes in the intra-tumoral and associated territories of kidney cancer. Cancer Cell. 2022;40:1583–1599.e1510. 36423636 10.1016/j.ccell.2022.11.001 PMC9767677

[16] Chen JK, Merrick KA, Kong YW, Izrael-Tomasevic A, Eng G, Handly ED, et al. An RNA damage response network mediates the lethality of 5-FU in colorectal cancer. Cell Rep. Med. 2024;5:101778. 39378883 10.1016/j.xcrm.2024.101778 PMC11514606

[17] Cui K, Wang K, Huang Z. Ferroptosis and the tumor microenvironment. J Exp Clin Cancer Res. 2024;43:315. 39614322 10.1186/s13046-024-03235-0 PMC11607824

[18] Ma RY, Black A, Qian BZ. Macrophage diversity in cancer revisited in the era of single-cell omics. Trends Immunol. 2022;43:546–63. 35690521 10.1016/j.it.2022.04.008

[19] Marabelle A, Le DT, Ascierto PA, Di Giacomo AM, De Jesus-Acosta A, Delord JP, et al. Efficacy of Pembrolizumab in Patients With Noncolorectal High Microsatellite Instability/Mismatch Repair-Deficient Cancer: Results From the Phase II KEYNOTE-158 Study. J Clin Oncol. 2020;38:1–10. 31682550 10.1200/JCO.19.02105 PMC8184060

[20] Lee HO, Hong Y, Etlioglu HE, Cho YB, Pomella V, Van den Bosch B, et al. Lineage-dependent gene expression programs influence the immune landscape of colorectal cancer. Nat Genet. 2020;52:594–603. 32451460 10.1038/s41588-020-0636-z

[21] Lu Y, Yang A, Quan C, Pan Y, Zhang H, Li Y, et al. A single-cell atlas of the multicellular ecosystem of primary and metastatic hepatocellular carcinoma. Nat Commun. 2022;13:4594. 35933472 10.1038/s41467-022-32283-3 PMC9357016

[22] Xu J, Fang Y, Chen K, Li S, Tang S, Ren Y, et al. Single-Cell RNA Sequencing Reveals the Tissue Architecture in Human High-Grade Serous Ovarian Cancer. Clin Cancer Res. 2022;28:3590–602. 35675036 10.1158/1078-0432.CCR-22-0296 PMC9662915

[23] Chen K, Wang Y, Hou Y, Wang Q, Long D, Liu X, et al. Single cell RNA-seq reveals the CCL5/SDC1 receptor-ligand interaction between T cells and tumor cells in pancreatic cancer. Cancer Lett. 2022;545:215834. 35917973 10.1016/j.canlet.2022.215834

[24] Heidegger I, Fotakis G, Offermann A, Goveia J, Daum S, Salcher S, et al. Comprehensive characterization of the prostate tumor microenvironment identifies CXCR4/CXCL12 crosstalk as a novel antiangiogenic therapeutic target in prostate cancer. Mol Cancer. 2022;21:132. 35717322 10.1186/s12943-022-01597-7 PMC9206324

[25] Jiang H, Yu D, Yang P, Guo R, Kong M, Gao Y, et al. Revealing the transcriptional heterogeneity of organ-specific metastasis in human gastric cancer using single-cell RNA Sequencing. Clin Transl Med. 2022;12:e730. 35184420 10.1002/ctm2.730 PMC8858624

[26] Pu W, Shi X, Yu P, Zhang M, Liu Z, Tan L, et al. Single-cell transcriptomic analysis of the tumor ecosystems underlying initiation and progression of papillary thyroid carcinoma. Nat Commun. 2021;12:6058. 34663816 10.1038/s41467-021-26343-3 PMC8523550

[27] Bischoff P, Trinks A, Obermayer B, Pett JP, Wiederspahn J, Uhlitz F, et al. Single-cell RNA sequencing reveals distinct tumor microenvironmental patterns in lung adenocarcinoma. Oncogene. 2021;40:6748–58. 34663877 10.1038/s41388-021-02054-3 PMC8677623

[28] Liberzon A, Subramanian A, Pinchback R, Thorvaldsdottir H, Tamayo P, Mesirov JP. Molecular signatures database (MSigDB) 3.0. Bioinformatics. 2011;27:1739–40. 21546393 10.1093/bioinformatics/btr260 PMC3106198

[29] Shigeoka T, Koppers M, Wong HH, Lin JQ, Cagnetta R, Dwivedy A, et al. On-site ribosome remodeling by locally synthesized ribosomal proteins in axons. Cell Rep. 2019;29:3605–3619.e3610. 31825839 10.1016/j.celrep.2019.11.025 PMC6915326

[30] Morral C, Stanisavljevic J, Hernando-Momblona X, Mereu E, Alvarez-Varela A, Cortina C, et al. Zonation of Ribosomal DNA Transcription Defines a Stem Cell Hierarchy in Colorectal Cancer. Cell Stem Cell. 2020;26:845–861.e812. 32396863 10.1016/j.stem.2020.04.012 PMC9006079

[31] Garden GA, Hartlage-Rubsamen M, Rubel EW, Bothwell MA. Protein masking of a ribosomal RNA epitope is an early event in afferent deprivation-induced neuronal death. Mol Cell Neurosci. 1995;6:293–310. 7496633 10.1006/mcne.1995.1023

[32] Hecht SS, Hatsukami DK. Smokeless tobacco and cigarette smoking: chemical mechanisms and cancer prevention. Nat Rev Cancer. 2022;22:143–55. 34980891 10.1038/s41568-021-00423-4 PMC9308447

[33] Joanito I, Wirapati P, Zhao N, Nawaz Z, Yeo G, Lee F, et al. Single-cell and bulk transcriptome sequencing identifies two epithelial tumor cell states and refines the consensus molecular classification of colorectal cancer. Nat Genet. 2022;54:963–75. 35773407 10.1038/s41588-022-01100-4 PMC9279158

[34] Dolezal JM, Dash AP, Prochownik EV. Diagnostic and prognostic implications of ribosomal protein transcript expression patterns in human cancers. BMC Cancer. 2018;18:275. 29530001 10.1186/s12885-018-4178-z PMC5848553

[35] Chu Y, Dai E, Li Y, Han G, Pei G, Ingram DR, et al. Pan-cancer T cell atlas links a cellular stress response state to immunotherapy resistance. Nat Med. 2023;29:1550–62. 37248301 10.1038/s41591-023-02371-y PMC11421770

[36] Chen Y, Wang D, Li Y, Qi L, Si W, Bo Y, et al. Spatiotemporal single-cell analysis decodes cellular dynamics underlying different responses to immunotherapy in colorectal cancer. Cancer Cell. 2024;42:1268–1285.e1267. 38981439 10.1016/j.ccell.2024.06.009

[37] Liu B, Hu X, Feng K, Gao R, Xue Z, Zhang S, et al. Temporal single-cell tracing reveals clonal revival and expansion of precursor exhausted T cells during anti-PD-1 therapy in lung cancer. Nat Cancer. 2022;3:108–21. 35121991 10.1038/s43018-021-00292-8

[38] Cheng S, Li Z, Gao R, Xing B, Gao Y, Yang Y, et al. A pan-cancer single-cell transcriptional atlas of tumor infiltrating myeloid cells. Cell. 2021;184:792–809.e723. 33545035 10.1016/j.cell.2021.01.010

[39] Sun L, Liu R, Wu ZJ, Liu ZY, Wan AH, Yan S, et al. Galectin-7 Induction by EHMT2 Inhibition enhances immunity in microsatellite stability colorectal cancer. Gastroenterology. 2024;166:466–82. 38065340 10.1053/j.gastro.2023.11.294

[40] Ghonim MA, Ibba SV, Tarhuni AF, Errami Y, Luu HH, Dean MJ, et al. Targeting PARP-1 with metronomic therapy modulates MDSC suppressive function and enhances anti-PD-1 immunotherapy in colon cancer. J Immunother Cancer. 2021;9:e001643. 33495297 10.1136/jitc-2020-001643 PMC7839867

[41] Wang JB, Li P, Liu XL, Zheng QL, Ma YB, Zhao YJ, et al. An immune checkpoint score system for prognostic evaluation and adjuvant chemotherapy selection in gastric cancer. Nat Commun. 2020;11:6352. 33311518 10.1038/s41467-020-20260-7 PMC7732987

[42] Aggarwal V, Workman CJ, Vignali DAA. LAG-3 as the third checkpoint inhibitor. Nat Immunol. 2023;24:1415–22. 37488429 10.1038/s41590-023-01569-z PMC11144386

[43] Zhang Q, Cui K, Yang X, He Q, Yu J, Yang L, et al. c-Myc-IMPDH1/2 axis promotes tumourigenesis by regulating GTP metabolic reprogramming. Clin Transl Med. 2023;13:e1164. 36629054 10.1002/ctm2.1164 PMC9832425

[44] Kofuji S, Hirayama A, Eberhardt AO, Kawaguchi R, Sugiura Y, Sampetrean O, et al. IMP dehydrogenase-2 drives aberrant nucleolar activity and promotes tumorigenesis in glioblastoma. Nat Cell Biol. 2019;21:1003–14. 31371825 10.1038/s41556-019-0363-9 PMC6686884

[45] Buffa FM, Harris AL, West CM, Miller CJ. Large meta-analysis of multiple cancers reveals a common, compact and highly prognostic hypoxia metagene. Br J Cancer. 2010;102:428–35. 20087356 10.1038/sj.bjc.6605450 PMC2816644

[46] Ye Y, Hu Q, Chen H, Liang K, Yuan Y, Xiang Y, et al. Characterization of hypoxia-associated molecular features to aid hypoxia-targeted therapy. Nat Metab. 2019;1:431–44. 31984309 10.1038/s42255-019-0045-8 PMC6980239

[47] Wang Z, Yang X, Liu C, Li X, Zhang B, Wang B, et al. Acetylation of PHF5A Modulates stress responses and colorectal carcinogenesis through alternative splicing-mediated upregulation of KDM3A. Mol Cell. 2019;74:1250–1263.e1256. 31054974 10.1016/j.molcel.2019.04.009

[48] Nelson RJ, Ziegelhoffer T, Nicolet C, Werner-Washburne M, Craig EA. The translation machinery and 70 kd heat shock protein cooperate in protein synthesis. Cell. 1992;71:97–105. 1394434 10.1016/0092-8674(92)90269-i

[49] Tian G, Hu C, Yun Y, Yang W, Dubiel W, Cheng Y, et al. Dual roles of HSP70 chaperone HSPA1 in quality control of nascent and newly synthesized proteins. EMBO J. 2021;40:e106183. 34010456 10.15252/embj.2020106183 PMC8246255

[50] Ali A, Garde R, Schaffer OC, Bard JAM, Husain K, Kik SK, et al. Adaptive preservation of orphan ribosomal proteins in chaperone-dispersed condensates. Nat Cell Biol. 2023;25:1691–703. 37845327 10.1038/s41556-023-01253-2 PMC10868727

[51] Kamada T, Togashi Y, Tay C, Ha D, Sasaki A, Nakamura Y, et al. PD-1(+) regulatory T cells amplified by PD-1 blockade promote hyperprogression of cancer. Proc Natl Acad Sci USA. 2019;116:9999–10008. 31028147 10.1073/pnas.1822001116 PMC6525547

[52] Zhu H, Chen J, Liu K, Gao L, Wu H, Ma L, et al. Human PBMC scRNA-seq-based aging clocks reveal ribosome to inflammation balance as a single-cell aging hallmark and super longevity. Sci Adv. 2023;9:eabq7599. 37379396 10.1126/sciadv.abq7599 PMC10306289

[53] Fakih M, Ouyang C, Wang C, Tu TY, Gozo MC, Cho M, et al. Immune overdrive signature in colorectal tumor subset predicts poor clinical outcome. J Clin Invest. 2019;129:4464–76. 31524634 10.1172/JCI127046 PMC6763253

[54] Weller C, Bartok O, McGinnis CS, Palashati H, Chang TG, Malko D, et al. Translation dysregulation in cancer as a source for targetable antigens. Cancer Cell. 2025;43:823–840.e818. 40154482 10.1016/j.ccell.2025.03.003 PMC12074880

[55] Galon J, Bruni D. Approaches to treat immune hot, altered and cold tumours with combination immunotherapies. Nat Rev Drug Discov. 2019;18:197–218. 30610226 10.1038/s41573-018-0007-y

[56] Kawashima SA, Chen Z, Aoi Y, Patgiri A, Kobayashi Y, Nurse P, et al. Potent, reversible, and specific chemical inhibitors of eukaryotic ribosome biogenesis. Cell. 2016;167:512–524.e514. 27667686 10.1016/j.cell.2016.08.070 PMC5116814

[57] Drygin D, Lin A, Bliesath J, Ho CB, O’Brien SE, Proffitt C, et al. Targeting RNA polymerase I with an oral small molecule CX-5461 inhibits ribosomal RNA synthesis and solid tumor growth. Cancer Res. 2011;71:1418–30. 21159662 10.1158/0008-5472.CAN-10-1728

[58] Hao Y, Hao S, Andersen-Nissen E, Mauck WM 3rd, Zheng S, Butler A, et al. Integrated analysis of multimodal single-cell data. Cell. 2021;184:3573–3587.e3529. 34062119 10.1016/j.cell.2021.04.048 PMC8238499

[59] McGinnis CS, Murrow LM, Gartner ZJ. DoubletFinder: Doublet Detection in Single-Cell RNA sequencing data using artificial nearest neighbors. Cell Syst. 2019;8:329–337.e324. 30954475 10.1016/j.cels.2019.03.003 PMC6853612

[60] Chen Y, Gong L, Cao Y, Liu Z, Wang Y, Cheng H, et al. Reprogramming tumor-associated macrophages by a dually targeted milk exosome system as a potent monotherapy for cancer. J Control Release. 2024;366:395–409. 38184235 10.1016/j.jconrel.2023.12.058

[61] Gong L, Tian L, Cui K, Chen Y, Liu B, Li D, et al. An off-the-shelf small extracellular vesicle nanomedicine for tumor targeting therapy. J Control Release. 2023;364:672–86. 37967724 10.1016/j.jconrel.2023.11.013

[62] Lv X, Lu X, Cao J, Luo Q, Ding Y, Peng F, et al. Modulation of the proteostasis network promotes tumor resistance to oncogenic KRAS inhibitors. Science. 2023;381:eabn4180. 37676964 10.1126/science.abn4180 PMC10720158

[63] Chasse H, Boulben S, Costache V, Cormier P, Morales J. Analysis of translation using polysome profiling. Nucleic Acids Res. 2017;45:e15. 28180329 10.1093/nar/gkw907 PMC5388431

[64] He Z, Brazovskaja A, Ebert S, Camp JG, Treutlein B. CSS: cluster similarity spectrum integration of single-cell genomics data. Genome Biol. 2020;21:224. 32867824 10.1186/s13059-020-02147-4 PMC7460789

[65] Andreatta M, Carmona SJ. UCell: Robust and scalable single-cell gene signature scoring. Comput Struct Biotechnol J. 2021;19:3796–8. 34285779 10.1016/j.csbj.2021.06.043 PMC8271111

[66] Hanzelmann S, Castelo R, Guinney J. GSVA: gene set variation analysis for microarray and RNA-seq data. BMC Bioinf. 2013;14:7. 10.1186/1471-2105-14-7 PMC3618321 23323831

[67] Cao J, Spielmann M, Qiu X, Huang X, Ibrahim DM, Hill AJ, et al. The single-cell transcriptional landscape of mammalian organogenesis. Nature. 2019;566:496–502. 30787437 10.1038/s41586-019-0969-x PMC6434952

