# Supplementary material for: Targeting ribosomes reprograms the tumour microenvironment and augments cancer immunotherapy
Source: Br J Cancer. 2025 Jul 11;133(6):756–70. doi: 10.1038/s41416-025-03109-y (PMC12449461; doi:10.1038/s41416-025-03109-y)
Supplement: Supplementary file 1 — Supplementary Information [file 41416_2025_3109_MOESM1_ESM.pdf]

Targeting ribosomes reprograms the tumour microenvironment and augments cancer immunotherapy

Supplementary Figures

Fig. S1 CX-5461 inhibits ribosome biogenesis in cancer cells (Related to Fig. 1). 2
Fig. S2 Integrate human Pan-cancer single-cell dataset across eight solid tumours (Related to Fig. 2)...3
Fig. S3 73-metagenes ribosomal state signature (Related to Fig. 2)..4
Fig. S4 Clinical outcomes of ribosomal state in human cancers (Related to Fig. 2). 7
Fig. S5 ScRNA-seq atlas of CD8$^+$ T cells (Related to Fig. 3). 8
Fig. S6 ScRNA-seq atlas of CD4$^+$ T cells (Related to Fig. 3). 9
Fig. S7 Immunotherapy ScRNA-seq atlas of T cells (Related to Fig. 4)...11
Fig. S8 ScRNA-seq atlas of macrophages (Related to Fig. 5)...13
Fig. S9 Targeting ribosome induces IC expressions (Related to Fig. 6)..15
Fig. S10 ScRNA-seq atlas of combined therapy (Related to Fig. 6). 16

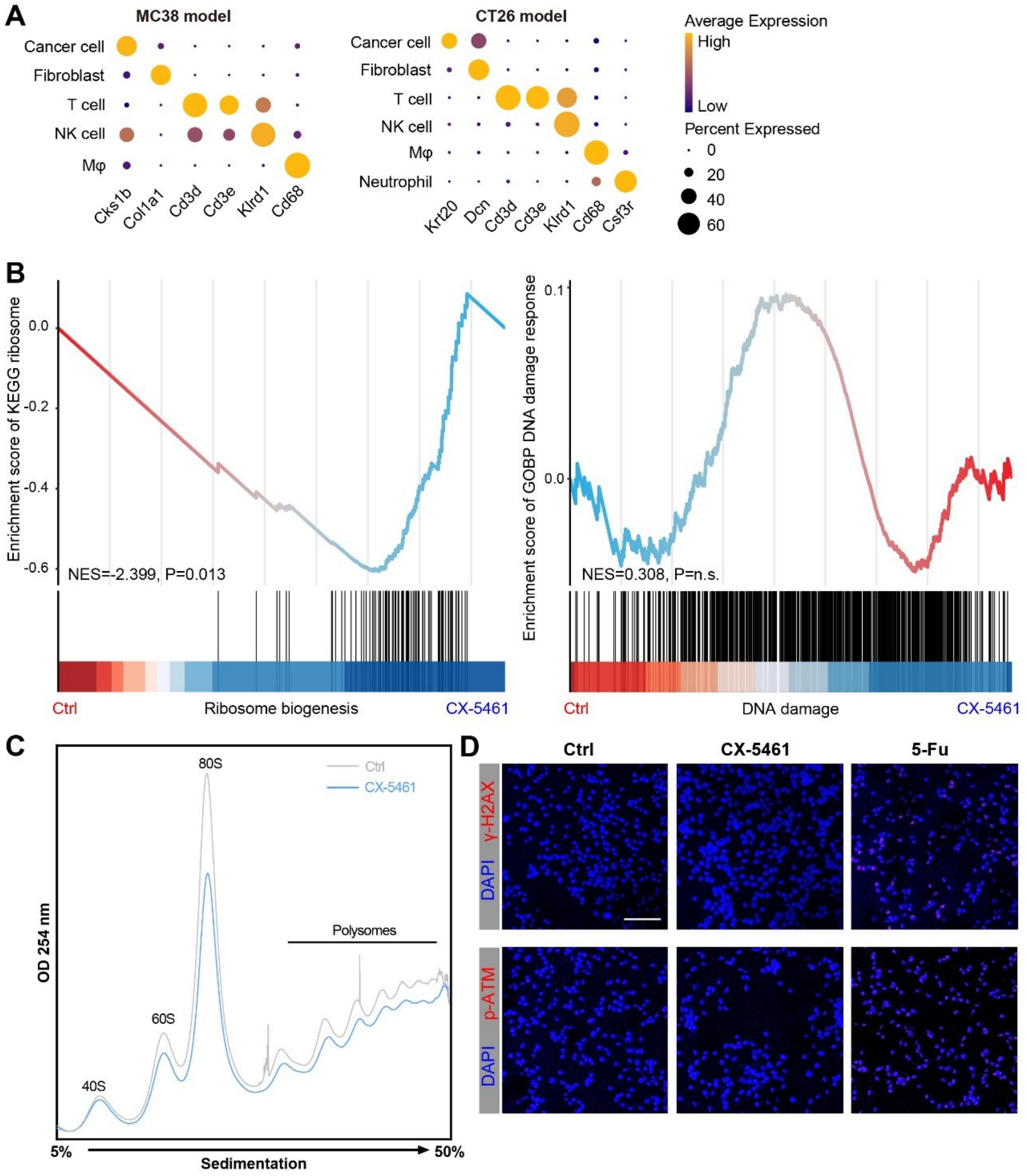

Fig. S1 CX-5461 inhibits ribosome biogenesis in cancer cells (Related to Fig. 1).

A. Dotplot showing expressions of marker genes of each cell type of CX-5461 administration in tumour murine models. Dot size indicates proportion of cells expressing genes, and dot color indicates mean expression of genes.

B. GSEA plot showing the enrichment of the ribosome biogenesis and DNA damage response signatures in cancer cells of CX-5461 administrated tumour murine models.

C. Polyribosome fractionation assays of RKO cells extract without or with CX-5461 administration.

D. Immunostaining of DNA damage marker γ-H2AX and p-ATM (all red) in CT26 cells upon CX-5461 or 5-FU treatment. Scale bar =100µm.

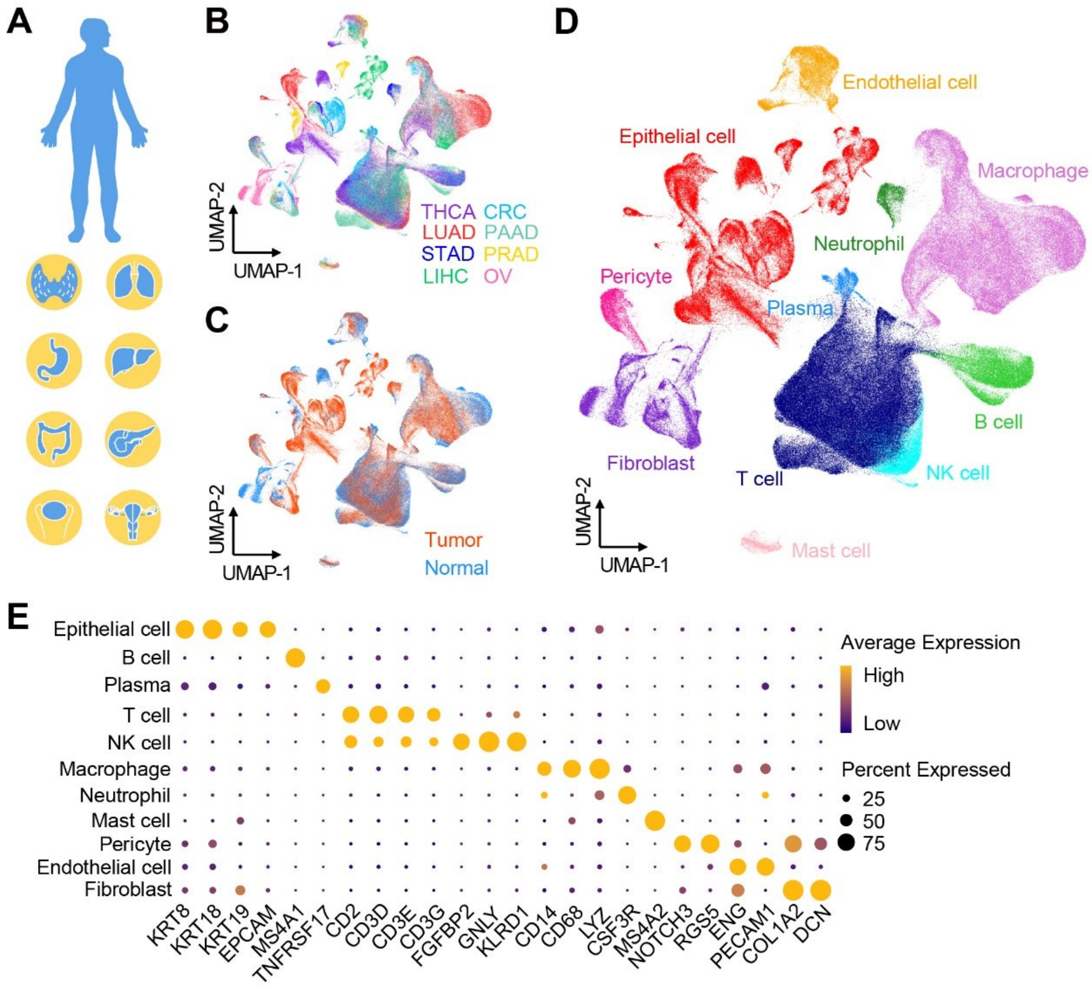

Fig. S2 Integrate human Pan-cancer single-cell dataset across eight solid tumours (Related to Fig. 2).

A. Human droplet-based integrated scRNA-seq data of eight solid tumour types we used in this study.

B-D. UMAP plots showing tumour (**B**), tissue (**C**) and cell (**D**) types of the integrated scRNA-seq data.

E. Dotplot showing expressions of marker genes of each cell type in integrated scRNA-seq data.

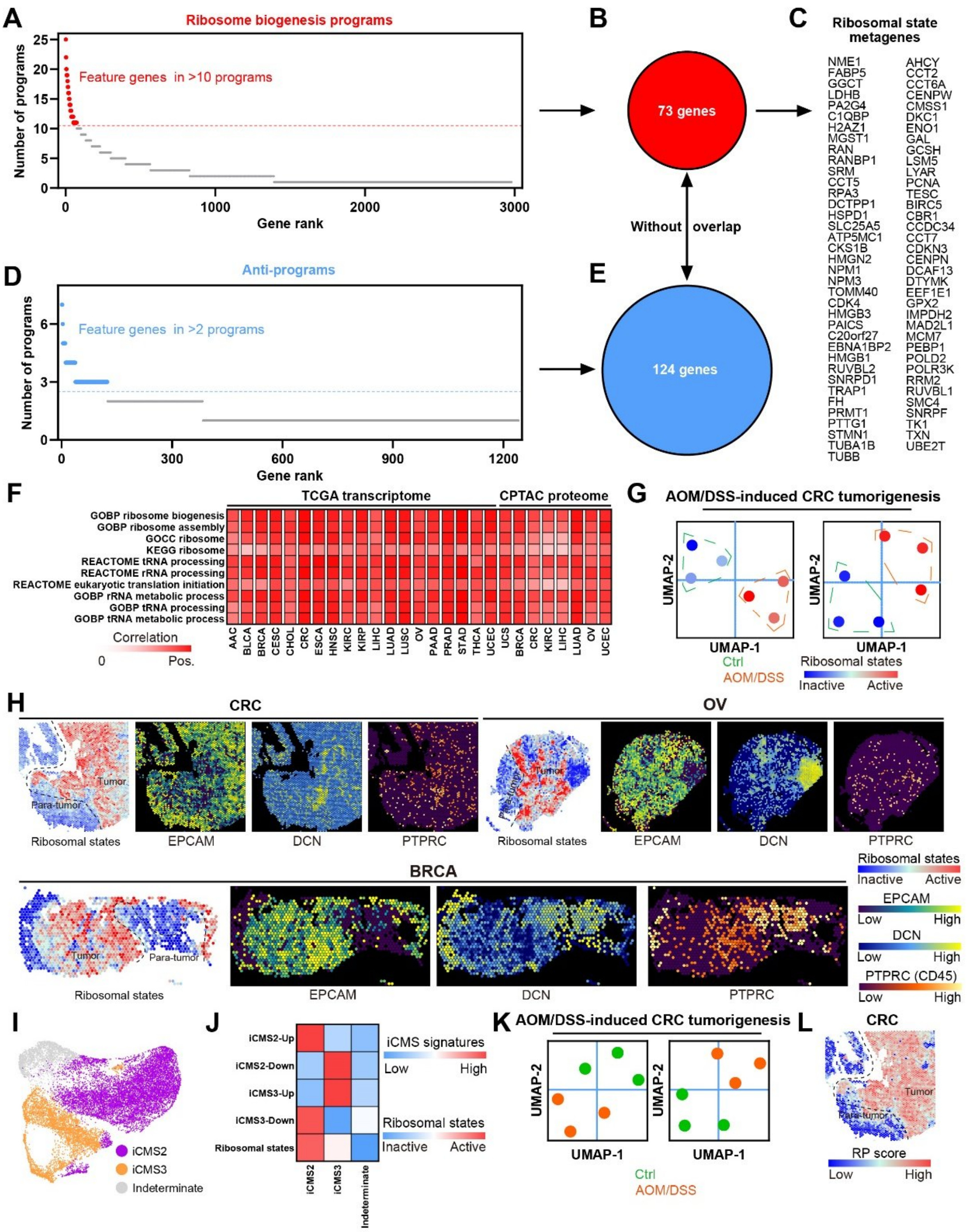

Fig. S3 73-metagenes ribosomal state signature (Related to Fig. 2).

A-B. The strategy for defining metagenes most frequently associated with ribosome-related programs.

C. 73 metagenes of the ribosomal state signature.

D-E. A test-negative control metagenes most frequently associated with anti-programs.

F. Heatmaps showing correlation coefficients between ribosome biogenesis levels and ribosomal assembly/rRNA/tRNA processes/translational activity in TCGA (mRNA) and CPTAC (protein) datasets.

G. Dotplots showing the ribosome biogenesis activity grouped by tissue types in AOM/DSS-induced CRC tumourigenesis from independent GEO datasets.

H. Visium images of CRC, OV (ovarian adenocarcinoma) and BRCA showing the spatial organization of spots assigned to cellular ribosome biogenesis, epithelial (EPCAM), stromal (DCN) and immune (PTPRC/CD45) cell markers.

I. UMAP plots showing iCMS types within CRC tumour epithelial cells of the integrated scRNA-seq data.

J. Heatmap showing levels of iCMS signatures and cellular ribosome biogenesis within CRC tumour epithelial cells of the integrated scRNA-seq data.

K. Dotplots showing tissue types in AOM/DSS-induced CRC tumourigenesis from independent GEO datasets based on ribosome protein genes expression.

L. Visium images of CRC showing the spatial organization of spots assigned to the ribosome protein (RP) score.

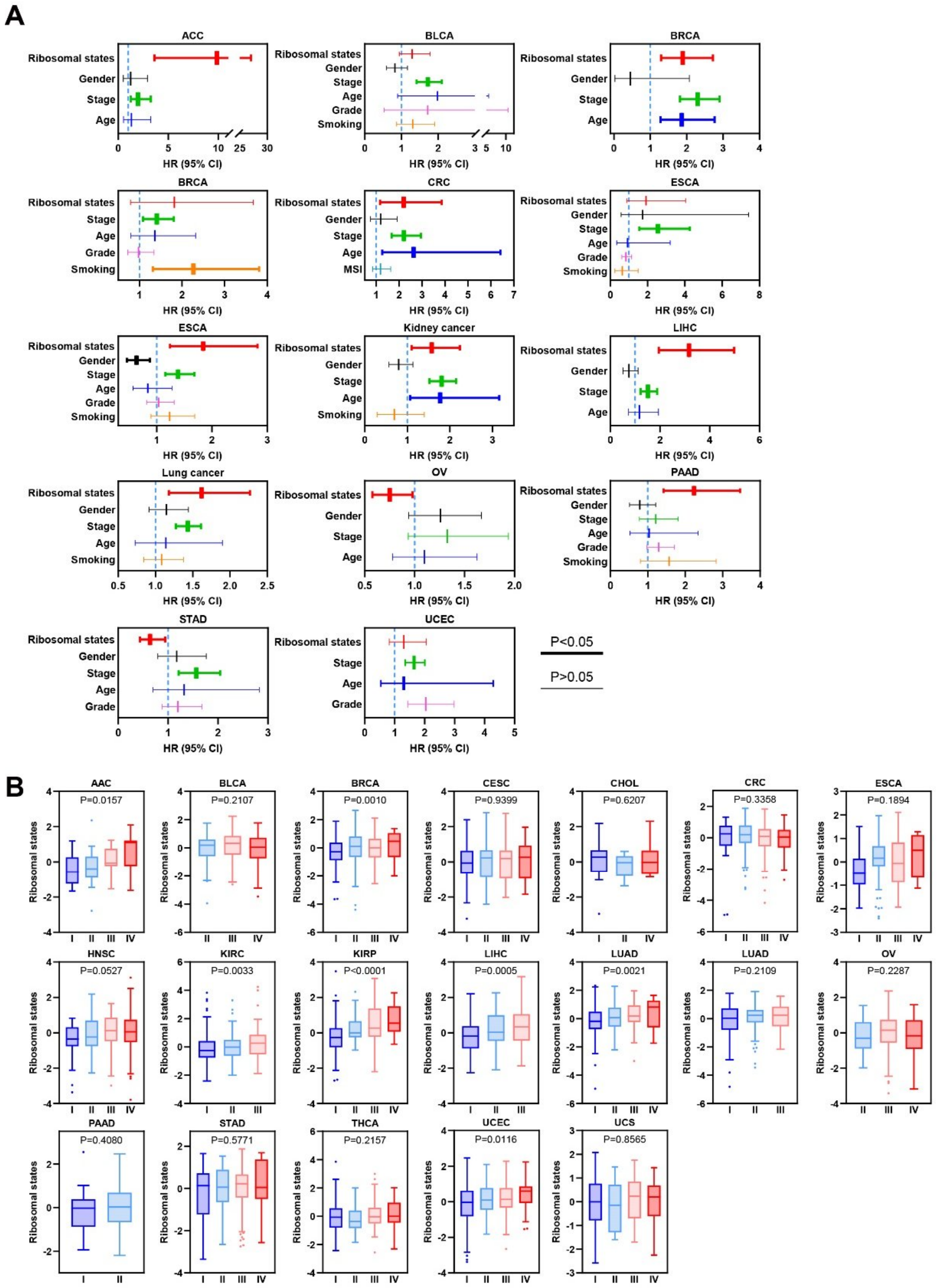
A
ACC
BLCA
BRCA
BRCA
CRC
ESCA
ESCA
Kidney cancer
LIHC
Lung cancer
OV
PAAD
STAD
UCEC
Ribosomal states
Gender
Stage
Age
Grade
Smoking
MSI
HR (95% CI)
P<0.05
P>0.05
B
AAC
P=0.0157
BLCA
P=0.2107
BRCA
P=0.0010
CESC
P=0.9399
CHOL
P=0.6207
CRC
P=0.3358
ESCA
P=0.1894
HNSC
P=0.0527
KIRC
P=0.0033
KIRP
P<0.0001
LIHC
P=0.0005
LUAD
P=0.0021
LUAD
P=0.2109
OV
P=0.2287
PAAD
P=0.4080
STAD
P=0.5771
THCA
P=0.2157
UCEC
P=0.0116
UCS
P=0.8565
Ribosomal states
I
II
III
IV

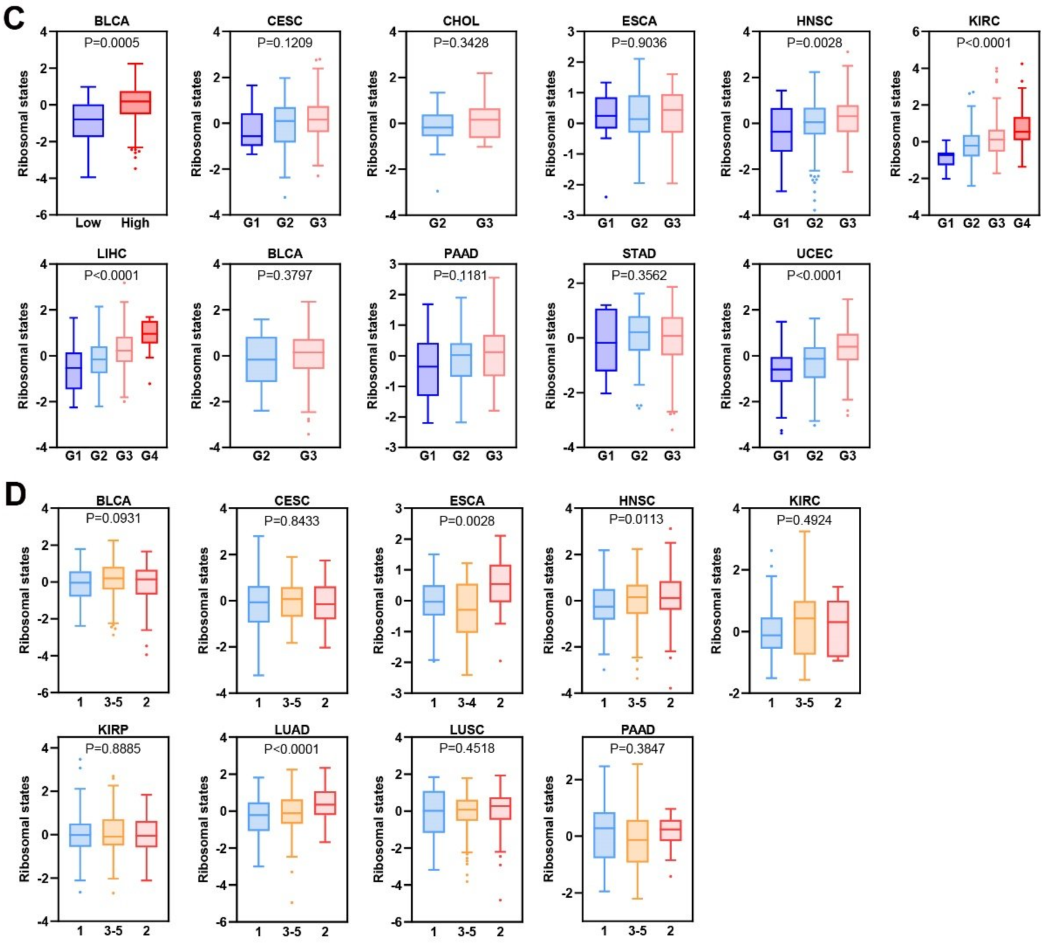

Fig. S4 Clinical outcomes of ribosomal state in human cancers (Related to Fig. 2).

A. Multivariate COX regression models showing the effect to overall survival of the ribosome biogenesis activity stratification and clinical indexes in the TCGA.

B-C. Boxplots showing the ribosome biogenesis activity grouped by stage (**B**) and grade (**C**) in the TCGA.

D. Boxplots showing the ribosome biogenesis activity grouped by smoking status in the TCGA. 1: Lifelong Non-smoker; 2: Current smoker; 3-5: Current reformed smoker. Boxplots show median, quartiles, min, and max.

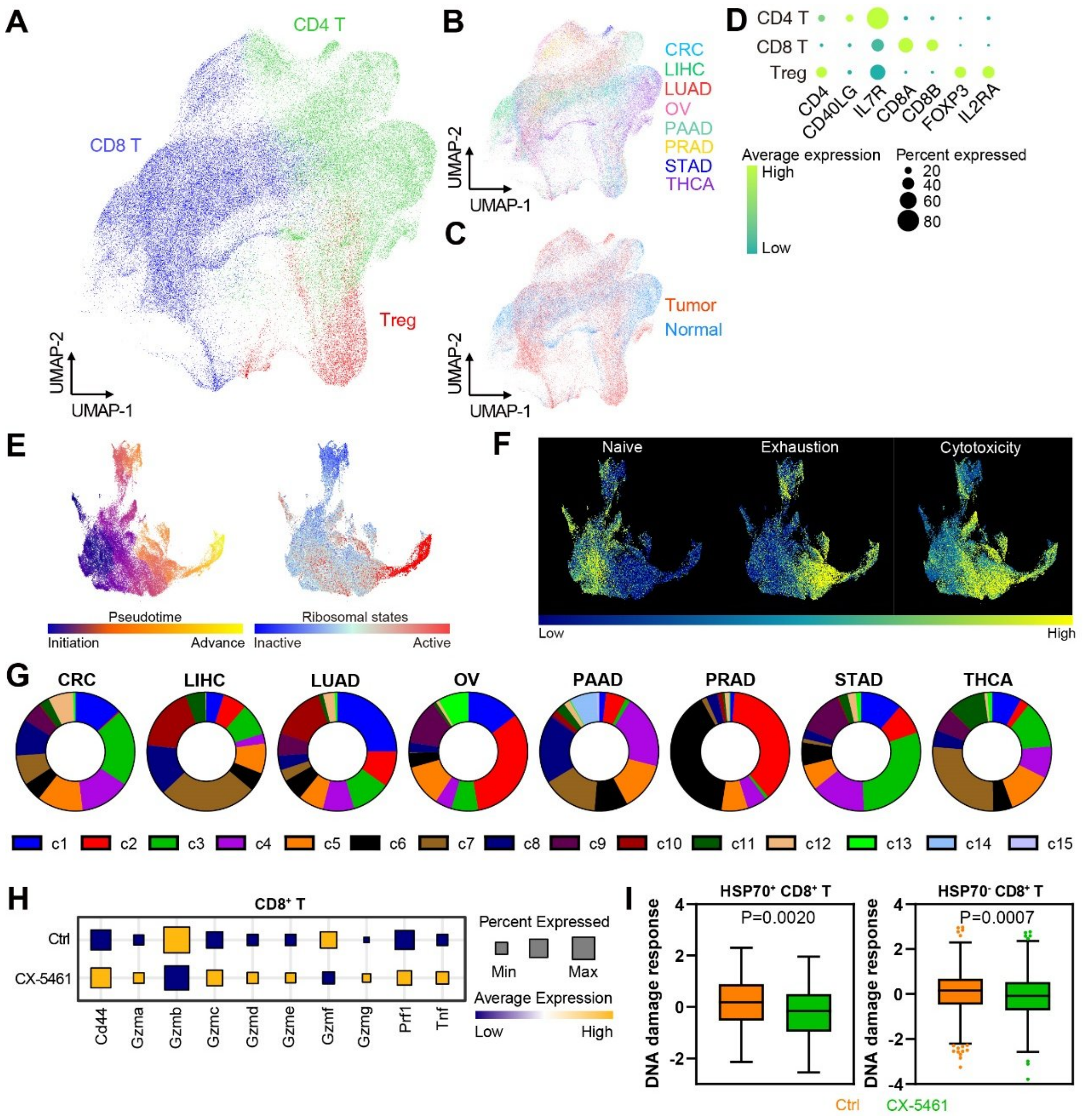

Fig. S5 ScRNA-seq atlas of CD8$^+$ T cells (Related to Fig. 3).

A-C. UMAP plots showing cell (**A**), tumour (**B**) and tissue (**C**) types within T cells.

D. Dotplot showing expression of canonical marker genes of each T cell subtype.

E-F. UMAP plots showing trajectory analysis of CD8$^+$ T cells. Cells are color coded for their corresponding pseudotime and cellular ribosome biogenesis (**E**) and CD8$^+$ T cell states distribution (**F**).

G. Pie plots showing proportions of each CD8$^+$ T cell cluster grouped by cancer types.

H. Dotplot showing expressions of activation and cytotoxic granules in CD8$^+$ T cells of CX-5461 administrated murine model. Dot size indicates proportion of cells expressing genes, dot color indicates mean expression of genes.

I. Boxplots showing DNA damage response in HSP70$^+$ CD8$^+$ T cells and HSP70$^-$ CD8$^+$ T cells of tumour murine models grouped by CX-5461 pre- and post-treatment samples.

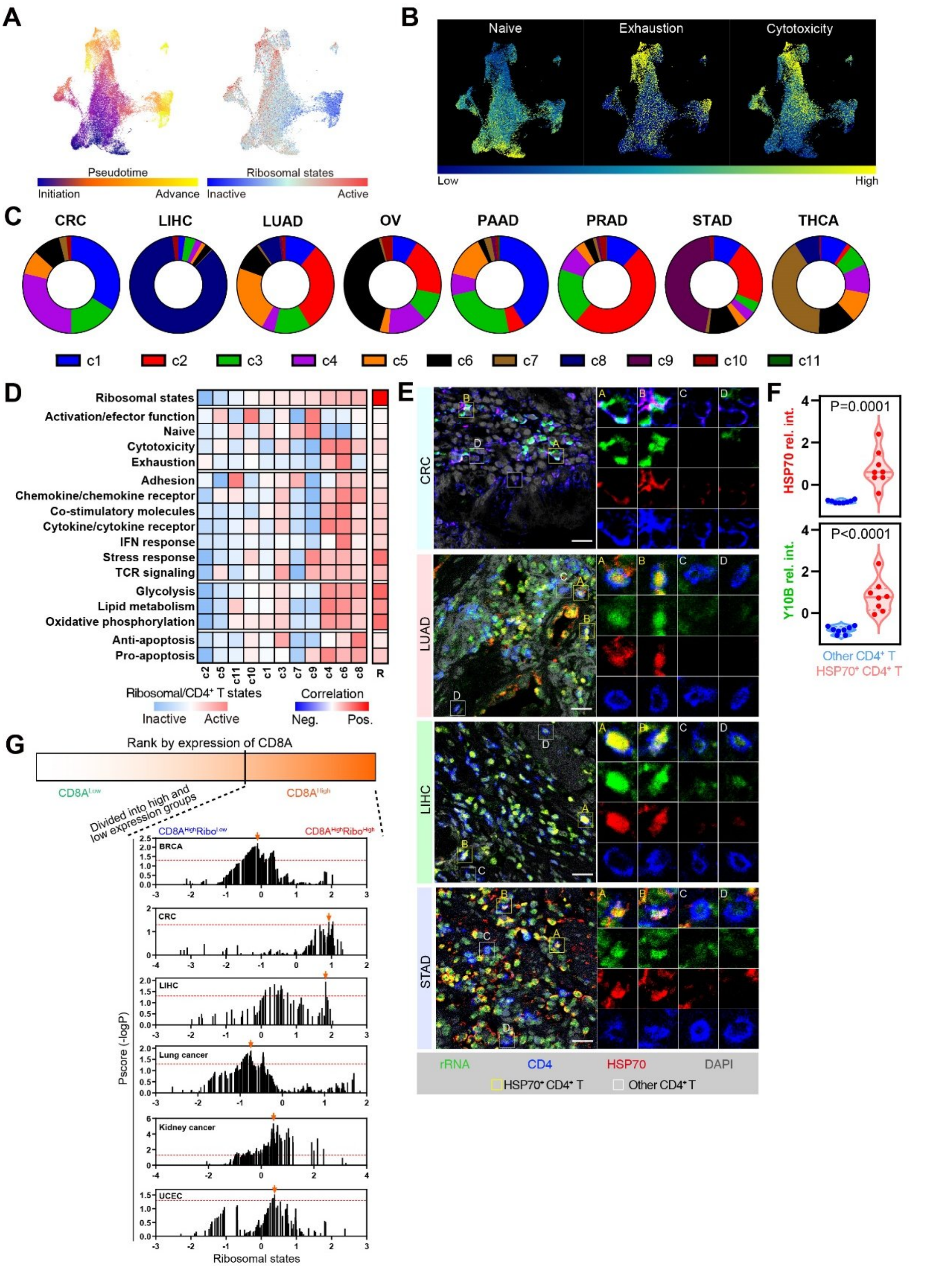

Fig. S6 ScRNA-seq atlas of CD4⁺ T cells (Related to Fig. 3).

A-B. UMAP plots showing trajectory analysis of CD4⁺ T cells. Cells are color coded for their corresponding pseudotime and cellular ribosome biogenesis (**A**) and CD4⁺ T cell states distribution (**B**).

C. Pie plots showing proportions of each CD4⁺ T cell cluster grouped by cancer types.

D. Heatmap showing the cellular ribosome biogenesis and 16 curated CD4⁺ T cell states across CD4⁺ T cell clusters. Right single- column heatmap showing the correlation levels between cellular ribosome biogenesis and 16 CD4⁺ T cell states.

E-F. Multicolor-immunostaining of rRNA (Y10B), $CD4^{+}$ T cells (CD4) and stress response marker (HSP70) in tissues of CRC, LIHC, LUAD and STAD. Scale bar for all immunostaining images = 20μm. The intensity of HSP70 and rRNA levels for each region (**F**). ROIs A and B represent $HSP70^{+}$ $CD4^{+}$ T (T_{str}), C and D represent $HSP70^{-}$ $CD4^{+}$ T cells.

G. Schema showing how the survival data were firstly processed and analyzed for the high and low expression of CD8A. Then $CD8A^{High}$ survival samples were ranked by expression and divided into high and low ribosomal states groups, examining differences Pscore (-logP).

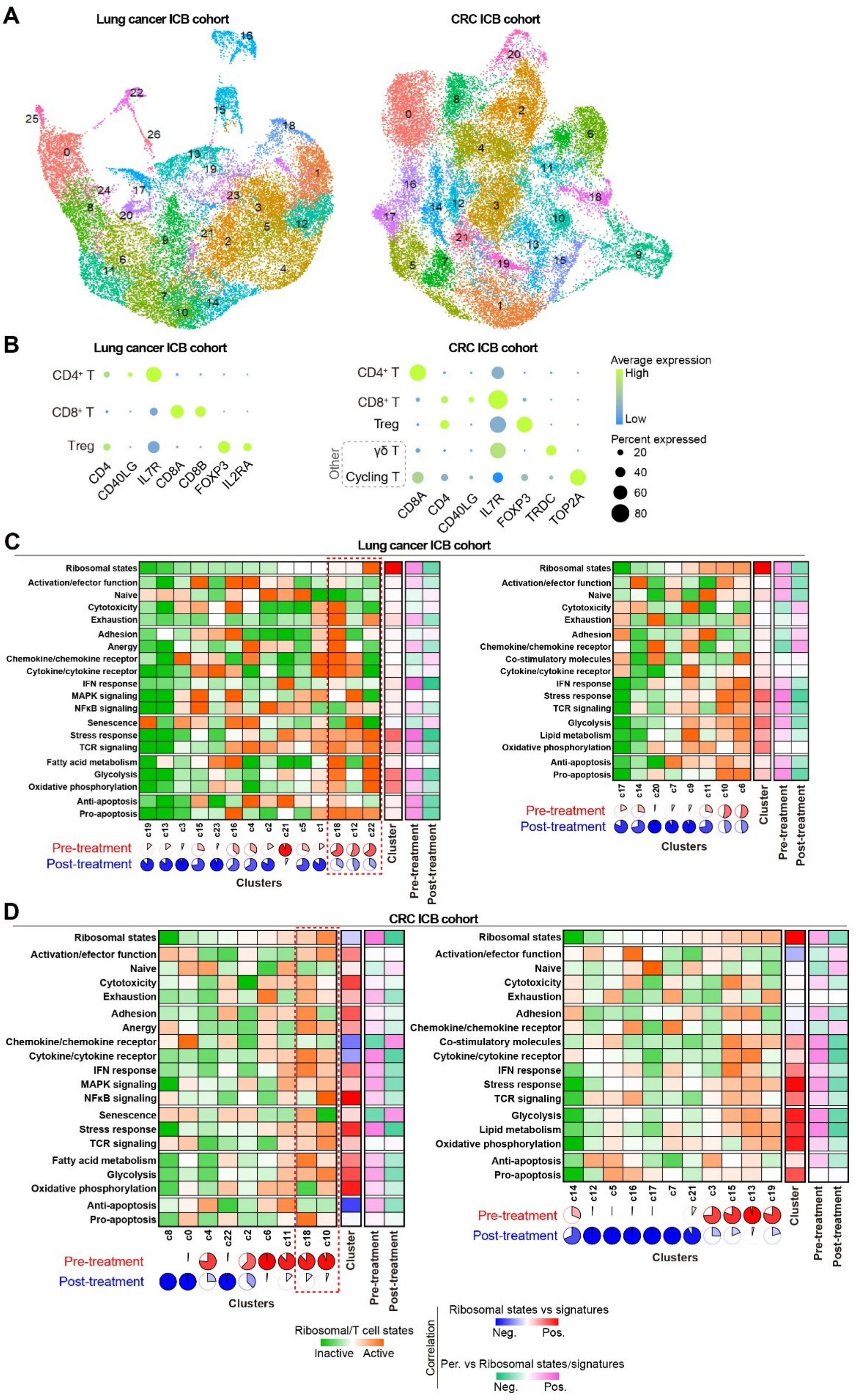

Fig. S7 Immunotherapy ScRNA-seq atlas of T cells (Related to Fig. 4).

A. UMAP plots showing clusters of T cells in lung cancer and CRC cases treated with ICB.

B. Dotplot showing expressions of marker genes of each T cell subtypes in lung cancer and CRC cases treatment of ICB. Dot size indicates proportion of cells expressing genes, dot color

indicates mean expression of genes.

C-D. Heatmaps showing the cellular ribosome biogenesis across CD8$^+$ T and CD4$^+$ T cell clusters in lung cancer (**C**) and CRC (**D**) cases treatment of ICB, as well as correlations between each T cell states and cellular ribosome biogenesis/treatment groups' distribution. Pie charts showing the distribution of pre- and post-treatment cell in cluster related to heatmap.

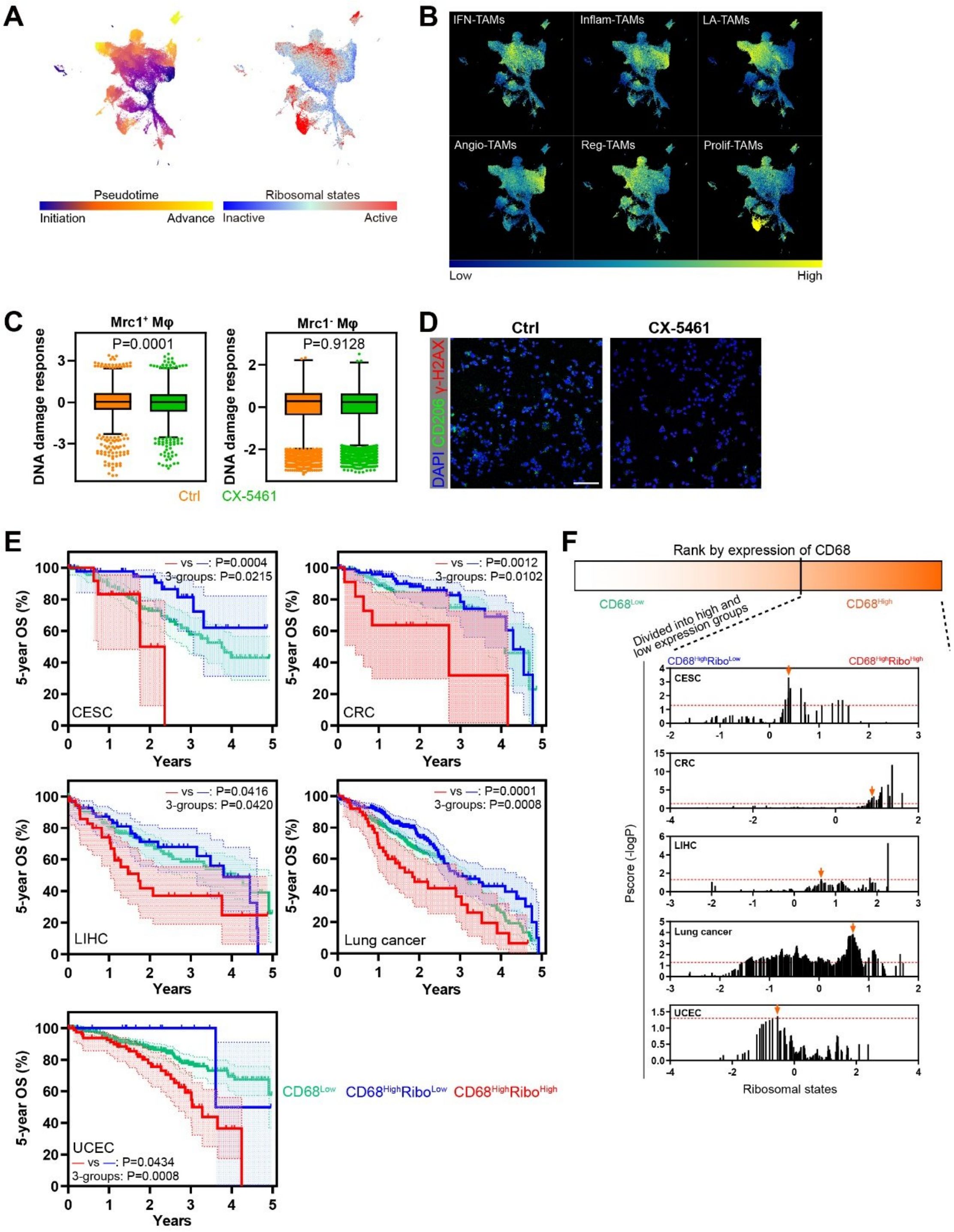

Fig. S8 ScRNA-seq atlas of macrophages (Related to Fig. 5).

A-B. UMAP plots showing trajectory analysis of TAMs. Cells are color coded for their corresponding pseudotime and cellular ribosome biogenesis (**A**) and TAM states distribution (**B**).

C. Boxplots showing DNA damage response in Mrc1^{+} Mφ and Mrc1^{-} Mφ of tumour murine models grouped by ctrl and CX-5461 treatment.

D. Immunostaining of DNA damage marker γ-H2AX (red) and anti-inflammatory/LA/Reg-TAM states marker CD206 (green) in immunosuppressive-like TAMs upon CX-5461 treatment. Immunosuppressive-like TAMs obtained from conditioned medium treated RAW264.7 cells.

Scale bar =100μm.

E. Kaplan–Meier plots showing the overall survival for each subgroup of the TAM infiltration stratified by ribosome biogenesis levels within CESC (cervical and endocervical carcinoma), CRC, LIHC (hepatocellular carcinoma), lung cancer and UCEC (endometrial carcinoma) of the TCGA.

F. Schema showing how the survival data were firstly processed and analyzed for the high and low expression of CD68. Then $CD68^{High}$ survival samples were ranked by expression and divided into high and low ribosomal states groups, examining differences Pscore (-logP).

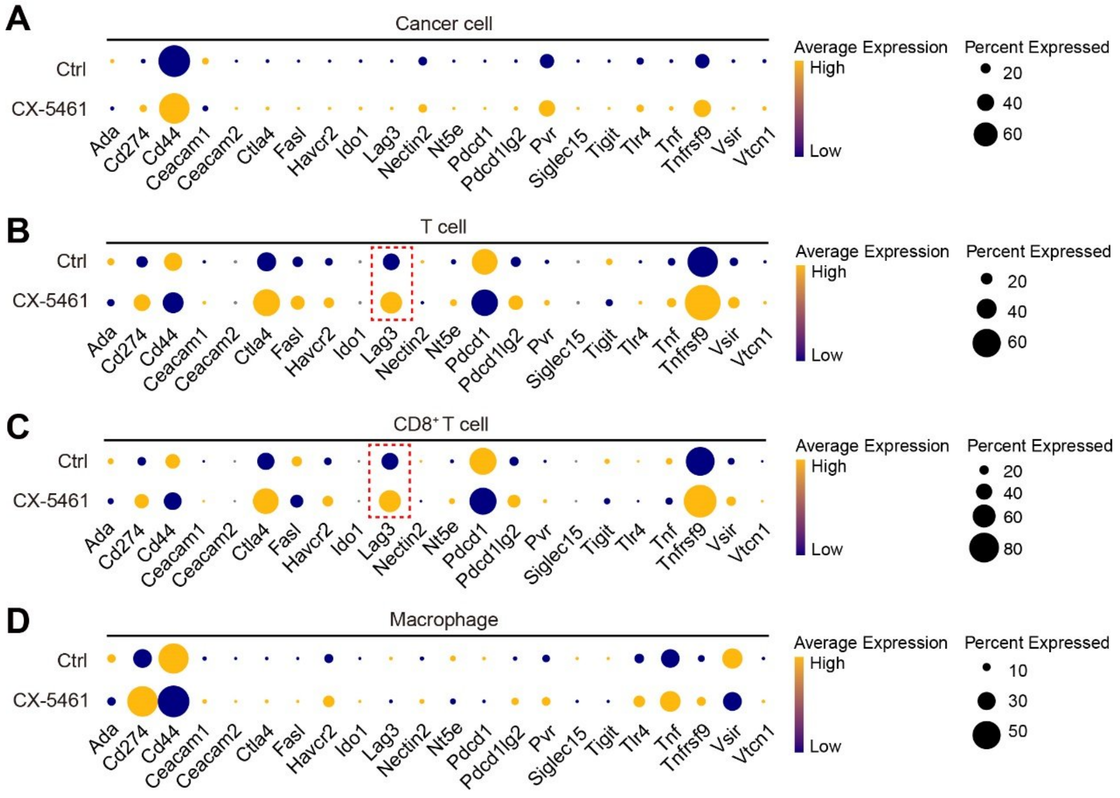

Fig. S9 Targeting ribosome induces IC expressions (Related to Fig. 6).

A-D. Dotplots showing expressions of immune checkpoint in cancer cells (**A**), T cells (**B**), CD8^{+} T cells (**C**) and macrophages (**D**) grouped by ctrl and CX-5461 treatment of CT26 murine models. Dot size indicates proportion of cells expressing genes, dot color indicates mean expression of genes.

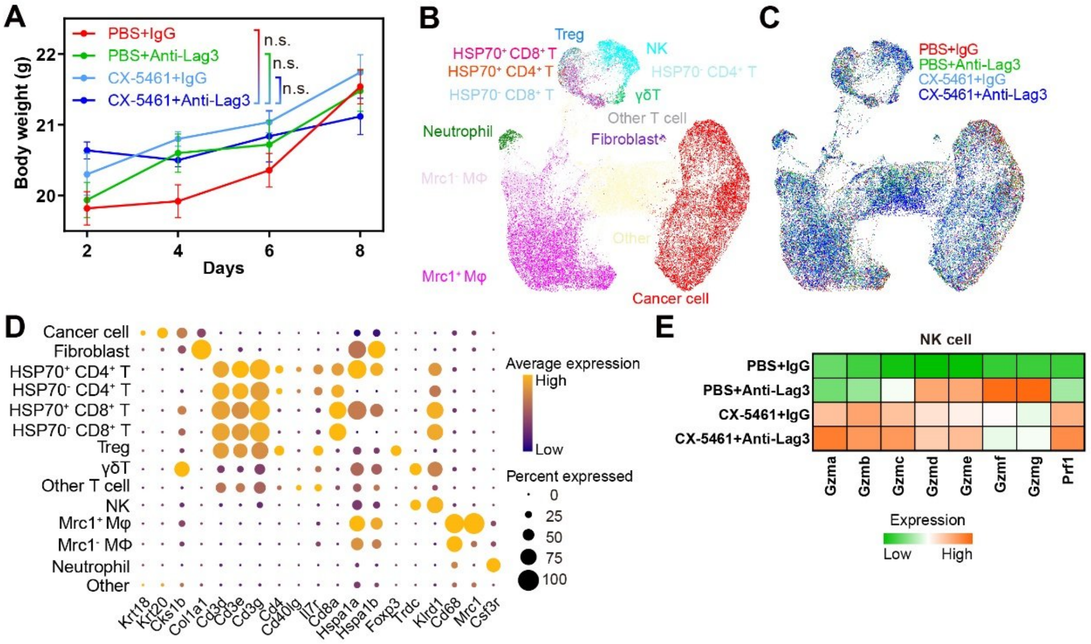

Fig. S10 ScRNA-seq atlas of combined therapy (Related to Fig. 6).

A. Body weight over time during therapy. Data are shown as means ± SEM.

B-C. UMAP plots showing cell types (**B**) and treatment groups (**C**) within samples of combined impact of CX-5461 and anti-Lag3 therapy of CT26 murine model.

D. Dotplot showing expressions of marker genes of each cell subtype in samples from (**B**). Dot size indicates proportion of cells expressing genes, dot color indicates mean expression of genes.

E. Heatmap showing expressions of cytotoxic granules in NK cells across treatment groups of CT26 murine model.
